# Quantitative Lipidomics and Spatial MS-Imaging Uncovered Neurological and Systemic Lipid Metabolic Pathways Underlying Troglomorphic Adaptations in Cave-Dwelling Fish

**DOI:** 10.1093/molbev/msac050

**Published:** 2022-03-12

**Authors:** Sin Man Lam, Jie Li, Huan Sun, Weining Mao, Zongmin Lu, Qingshuo Zhao, Chao Han, Xia Gong, Binhua Jiang, Gek Huey Chua, Zhenwen Zhao, Fanwei Meng, Guanghou Shui

**Affiliations:** 1 State Key Laboratory of Molecular Developmental Biology, Institute of Genetics and Developmental Biology, Chinese Academy of Sciences, Beijing, China; 2 LipidALL Technologies Company Limited, Changzhou, Jiangsu Province, China; 3 University of Chinese Academy of Sciences, Beijing, China; 4 Qujing Aquaculture Station, Qujing, Yunan Province, China; 5 Chinese Academy of Sciences, Institute for Stem Cell and Regeneration, Beijing, China; 6 State Key Laboratory of Membrane Biology, Institute of Zoology, Chinese Academy of Sciences, Beijing, China; 7 Beijing National Laboratory for Molecular Sciences, CAS Research/Education Center for Excellence in Molecular Sciences, Key Laboratory of Analytical Chemistry for Living Biosystems, Institute of Chemistry Chinese Academy of Sciences, Beijing Mass Spectrum Center, Beijing, China

**Keywords:** lipidomics, mass-spectrometry imaging, cavefish, lipid metabolism, docosahexaenoic acid, arachidonic acid, oxidative phosphorylation

## Abstract

*Sinocyclocheilus* represents a rare, freshwater teleost genus endemic to China that comprises the river-dwelling surface fish and the cave-dwelling cavefish. Using a combinatorial approach of quantitative lipidomics and mass-spectrometry imaging (MSI), we demonstrated that neural compartmentalization of lipid distribution and lipid metabolism is associated with the evolution of troglomorphic traits in *Sinocyclocheilus*. Attenuated docosahexaenoic acid (DHA) biosynthesis via the Δ4 desaturase pathway led to reductions in DHA-phospholipids in cavefish cerebellum. Instead, cavefish accumulates arachidonic acid-phospholipids that may disfavor retinotectal arbor growth. Importantly, MSI of sulfatides coupled with immunostaining of myelin basic protein and transmission electron microscopy images of hindbrain axons revealed demyelination in cavefish raphe serotonergic neurons. Demyelination in cavefish parallels the loss of neuroplasticity governing social behavior such as aggressive dominance. Outside the brain, quantitative lipidomics and qRT-PCR revealed systemic reductions in membrane esterified DHAs in the liver, attributed to suppression of genes along the Sprecher pathway (*elovl2*, *elovl5*, and *acox1*). Development of fatty livers was observed in cavefish; likely mediated by an impeded mobilization of storage lipids, as evident in the diminished expressions of *pnpla2*, *lipea*, *lipeb*, *dagla*, and *mgll*; and suppressed β-oxidation of fatty acyls via both mitochondria and peroxisomes as reflected in the reduced expressions of *cpt1ab*, *hadhaa*, *cpt2*, *decr1,* and *acox1*. These neurological and systemic metabolic adaptations serve to reduce energy expenditure, forming the basis of recessive evolution that eliminates nonessential morphological and behavioral traits and giving cavefish a selective advantage to thrive in caves where proper resource allocation becomes a major determinant of survival.

## Introduction

*Sinocyclocheilus* (Cypriniformes, Cyprinidae) is a rare, freshwater teleost genus endemic to Southwestern China, one of world’s largest cave-rich karst geomorphologic regions (Meng et al. 2013). *Sinocyclocheilus* can exist in different forms, predominantly the surface-dwelling species and the cave-dwelling species (Meng et al. 2013; Zhao et al. 2021). *Sinocyclocheilus* cave dwellers first colonized cave habitats to seek refuge in deeper waters in response to widespread drying associated with the aridification of China during the late Miocene and Pliocene (Mao et al. 2021). Cave habitat is considered as an extreme environment due to perpetual darkness and food scarcity (Simon et al. 2017). Cave-dwelling fish across the world independently evolved a series of troglodyte characteristics and behavioral adaptations to enhance survival, such as enhanced sensation, eyesight degeneration, loss of pigmentation, and dominance aggressiveness, as well as a disrupted circadian rhythm (Elipot et al. 2013, 2014; Meng et al. 2013; Soares and Niemiller 2013). As the brain of vertebrates displays a high degree of conservation both in terms of anatomical structures and neuromodulatory signaling (Katz and Lillvis 2014), rapid evolution of behavior may instead rely on distinct compartmentalization of neuromodulatory signaling networks (Chattopadhyay et al. 2012). Lipids, as biophysical constituents of biological membranes, partake in the morphological and functional compartmentalization in the brain via membrane remodeling (Aureli et al. 2015). For example, the formation and remodeling of myelin sheaths around specific axons can be harnessed to fine-tune neural plasticity in achieving bidirectional regulation of functional behavior (Chang et al. 2016). The surface fish and cavefish thus provide a natural setting for understanding how brain lipid metabolism may regulate neural plasticity leading to distinct behavioral traits during evolution.

Serotonin (5-HT) signaling mediated by different subtypes of 5-HT receptors (5-HTR) is phylogenetically conserved. Aberrant 5-HT signaling has been implicated in various neurological diseases including depression and schizophrenia (Bjork et al. 2010). Differential regulation of serotonergic signaling in specific neuronal populations was found to constitute the basis of differential behavioral patterns in Mexican surface fish and cavefish (Elipot et al. 2013). Enhanced hypothalamic serotoninergic signaling in cavefish mediates passive foraging behavior, although downregulation of raphe serotoninergic signaling modulates social dominance and aggressive behavior in river-dwelling surface fish (Elipot et al. 2013). In a later study, it was found that a mutation in monoamine oxidase (MAO) leads to impeded 5-HT turnover and thus higher 5-HT content throughout the brain of cavefish, partly explaining why cavefish are unable to downregulate raphe serotonergic signaling to establish dominance aggressiveness like their surface counterparts (Elipot et al. 2014).This does not explain, however, why surface fish can elicit experience-dependent downregulation in raphe 5-HT signaling. Since membrane lipid micro milieu can possibly alter receptor ligand-binding efficiency and its subsequent activity (Sjögren and Svenningsson 2007), dynamic changes in neural lipid membrane remodeling may offer a quick avenue for modulating 5-HT signal transduction.

In this study, we investigated region-specific differences in the spatial distribution and metabolism of lipids in the brain, in association with distinct behaviors of the *Sinocyclocheilus* cavefish (*Sinocyclocheilu anophthalmus*) and surface fish (*Sinocyclocheilu augustiporus*). We leveraged on a combination of high-sensitivity targeted mass spectrometry based on multiple reaction monitoring and matrix-assisted laser desorption ionization-Fourier-transform ion cyclotron resonance mass spectrometry (MALDI-FTICR MS) on adjacent brain tissue sections to obtain a spatial atlas of lipid distribution in four major brain regions of *Sinocyclocheilus* species. Investigating these differences can help one to understand how lipids modulate neuroplasticity and behavioral traits and confers new evidence that neural compartmentalization of lipid distribution and lipid metabolism may function to regulate behavior in *Sinocyclocheilus* species. In addition to the brain, we also examined quantitative lipidome changes in the eyes and livers of the two fish species, uncovering alterations in systemic metabolism that facilitate troglomorphic adaptations to life in cave environments. In all, the neurological and systemic adaptations in cavefish follow a common theme to eliminate nonessential morphological and behavioral traits, such as the loss of advanced eye functions and social behavior, which cumulatively serve to reduce energy expenditure and confer a selective advantage to surviving in cave environments with irregular food supply.

## Results

### Increased Oxidative Phosphorylation in the Brain of Cavefish

**Fig. 1.**
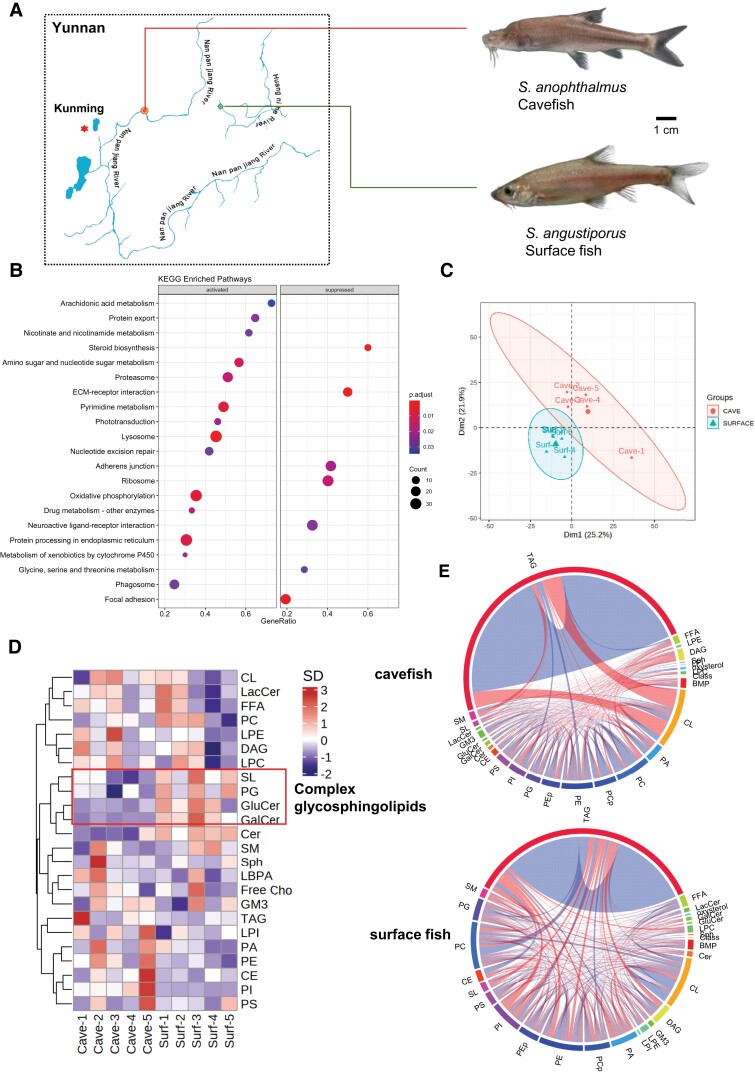
Changes in whole-brain transcriptome and lipidome between cavefish and surface fish. (*A*) Schematic illustration on river drainages near the collection sites of cavefish ( *Sinocyclocheilus anopthalmus*) (orange mark) and surface fish (*S. angustiporus*) (green marks) near the city of Kunming, Yunnan province. Scale bar: 1 cm. (*B*) Enrichment plot illustrates top dysregulated pathways based on whole-brain transcriptomics analysis from Meng et al. (2013, 2018) on the brains of cavefish relative to surface fish. Gene ratio denotes the number of differentially expressed genes relative to the total number of genes under the specific term. (*C*) Principal component analysis based on whole-brain lipidome of surface fish and cavefish. *n* = 5 biological replicates for each fish species. (*D*) Hierarchical clustering heatmap illustrates patterns of changes in major lipid classes between the brains of cavefish and surface fish. Box indicates complex glycosphingolipids including sulfatides (SL), glucosylceramide (GluCer), and galactosylceramide (GalCer) were appreciably upregulated in the brains of surface fish. *n* = 5 biological replicates for each fish species. (*E*) Chord diagrams show the changes in lipid correlations in the brain of cavefish and surface fish, respectively. Lipid correlations were calculated by Spearman correlation analyses with cutoffs in correlation coefficients at *≥*0.7 and *P* < 0.05. Band width indicates the number of correlations and color indicates direction of correlation (red: negative correlation; blue: positive correlation). *n* = 5 biological replicates for each fish species.

*Sinocyclocheilus* fishes (declared as national second-class protected animals in China in 2020) were wild-caught near Kunming, Yunnan province of China between the years of 2016 and 2018. The cave species, *S. anophthalmus*, were found in a cave along the Nanpanjiang River (supplementary fig. 1, Supplementary Material online), whereas the surface fish, *S. angustiporus*, were obtained at sites along the Huangnihe River (fig. 1*A*). Akin to *Astyanax mexicanus*, we found that the surface species of *Sinocyclocheilus* displays obvious aggregation behavior in the wild and in the laboratory tank, whereas the cavefish *S. anophthalmus* behave as a single individual in the cave and in a dispersed state in the fish tank (supplementary fig. 1, Supplementary Material online). Using whole-brain transcriptome data published previously (Meng et al. 2013, 2018) (supplementary table S1, Supplementary Material online), we first performed Gene Set Enrichment Analysis (GSEA) to investigate the differences in KEGG pathways between these two species. GSEA revealed enhanced arachidonic acid (ARA) metabolism and oxidative phosphorylation in the brain of cavefish relative to surface fish (fig. 1*B*). We then analyzed the whole-brain lipidomes (comprising >750 individual lipids) (supplementary table S2, Supplementary Material online) of the two species using targeted LC-MS/MS approaches (Song, et al. 2020; Lam, et al. 2021). Principal component analysis showed that the brain lipidomes of cavefish and surface fish were clearly segregated (fig. 1*C*), and the clustering heatmap of major lipid classes illustrated appreciable elevations of complex glycosphingolipids in the brains of surface fish (fig. 1*D*). To examine global alterations in lipid coregulations, we analyzed lipid correlations in the whole-brain lipidome of cavefish and surface fish, respectively (fig. 1*E*). Chord diagrams revealed a strong negative correlation (red shades) between storage triacylglycerols (TAGs) and mitochondria-resident cardiolipins (CLs) in the brain of cavefish, which was absent in surface fish. CLs denote the signature phospholipid class of the mitochondria, which are localized at the foldings of the inner mitochondrial membrane, known as crista—the primary site of oxidative phosphorylation where protein components of the electron transport chains reside (Ikon and Ryan 2017). A negative correlation, therefore, indicates high CLs at the expense of storage TAGs, implying an enhanced mobilization of storage TAGs into free fatty acyls, which are consumed via mitochondrial oxidative phosphorylation in cavefish brain (Ikon and Ryan 2017). Lipid correlation analyses thus corroborated pathway analysis based on GSEA that cavefish brains elicit enhanced oxidative phosphorylation. Indeed, inhibition of brain oxidative phosphorylation was previously shown to increase aggressive behavior in honeybees and fruit flies (Li-Byarlay et al. 2014). Results based on the *Sinocyclocheilus* cavefish and surface fish thus support the preceding finding that enhanced neural oxidative phosphorylation is negatively associated with aggressive behavior.

### Cavefish and Surface Fish Displayed Opposite Neural Patterning of DHA and ARA

**Fig. 2.**
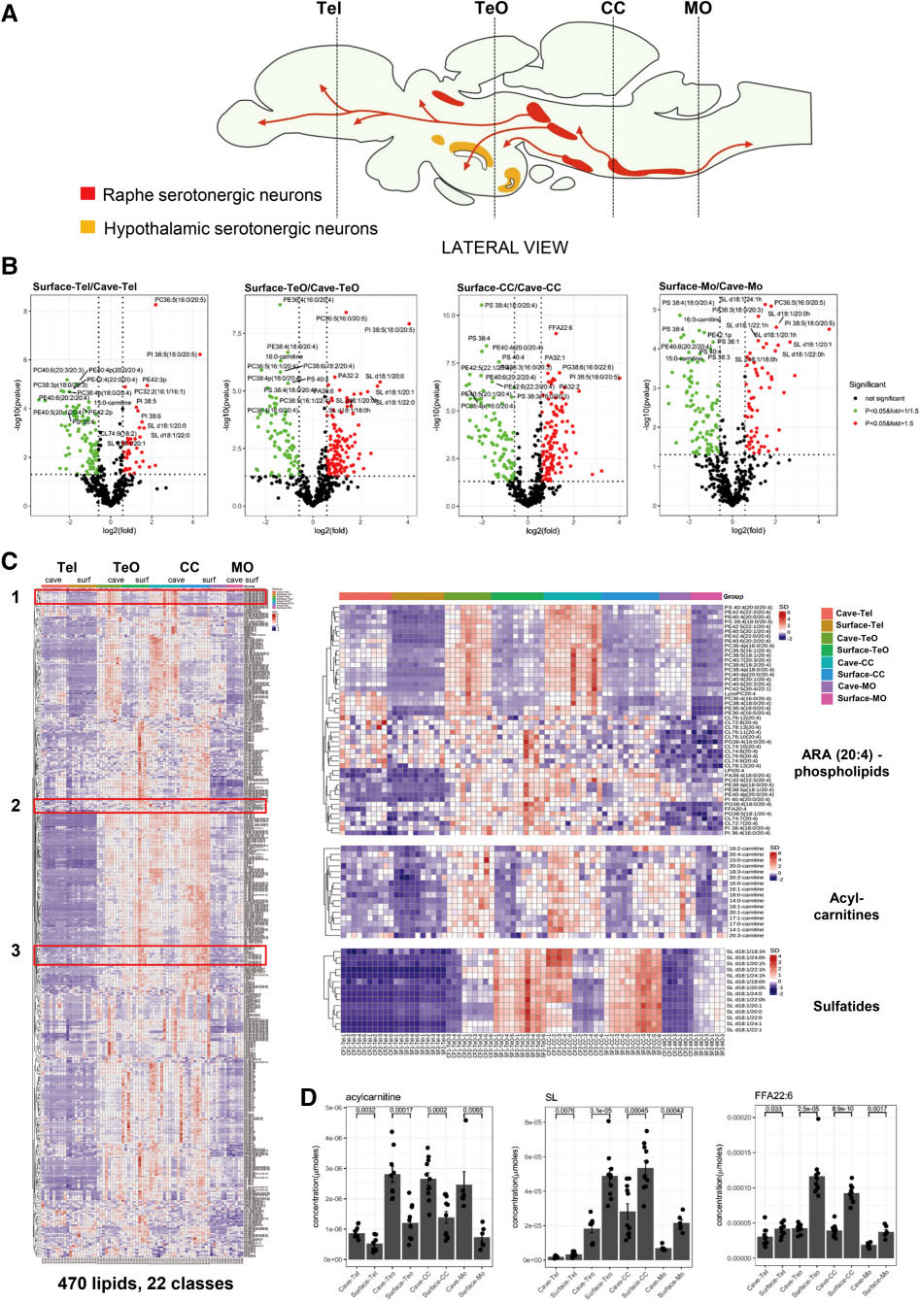
Region-specific changes in lipidomes across four brain regions of cavefish and surface fish. Tel, telencephalon; TeO, tectum opticum; CC, corpus cerebelli; MO, medulla oblongata. (*A*) Schematic illustration of the lateral and ventral views of serotonergic neuron populations (regions colored yellow and red) in the brain of teleosts adapted from Prasad et al. (2015) and Elipot et al. (2013). Four distinct regions laterally span the teleost’s brain, which includes the telencephalon (Tel), the midbrain region largely covered by the tectum opticum (TeO), the hindbrain/brain stem region rostrally covered by the cerebellum (CC), and the medulla oblongata (MO) that grades into the spinal cord. The major serotonergic populations in the brain of teleost include the hypothalamic populations (yellow) and raphe populations (red). Most teleosts preserve two l-tryptophan hydroxylases (TPHs) for biosynthesis of serotonin from l-tryptophan. The raphe serotonergic populations (red) utilize TPH2 for serotonin production, whereas the hypothalamic populations rely on TPH1 (yellow). The superior raphe nuclei extend into the forebrain and midbrain, whereas the inferior raphe populations project into the hindbrain-spinal cord region (MO). (*B*) Volcano plots display top lipids that were most significantly different in each pairwise comparison between surface fish and cavefish brain sections from each brain region, based on magnitudes of *P*-value and fold-changes. Two-sided Welch’s *t*-test was used for pairwise comparisons. *n* = 3–4 brain sections from three biological replicates for Tel, TeO, and CC; *n* = 2 brain sections from three biological replicates for MO. (*C*) Hierarchical clustering was performed to aggregate lipid classes exhibiting comparable patterns of change across four regions (Tel, TeO, CC, and MO) between the brains of cavefish and surface fish. Patterns were visually examined and three major clusters were selected and expanded on the right, which included arachidonyl (ARA)-phospholipids, acylcarnitines, and sulfatides (SL). *n* = 3–4 brain sections from three biological replicates for Tel, TeO, and CC, *n* = 2 brain sections from three biological replicates for MO. Please refer to supplementary table S4, Supplementary Material online for the comprehensive list of lipids ordered by hierarchical clustering (arranged from top to bottom of the heatmap). (*D*) Barplots on changes in total acylcarnitine, sulfatides (SL), and docosahexaenoic acid (FFA 22:6) across Tel, TeO, CC, and MO in cavefish versus surface fish. *P*-values from two-sided Welch’s *t*-test for each pairwise comparison were illustrated. *n* = 3–4 brain sections from three biological replicates for Tel, TeO, and CC, *n* = 2 brain sections from three biological replicates for MO.

To elucidate region-specific differences in lipid metabolism and spatial lipid distribution, we then systematically sectioned the whole-brain of each species transversely. The brain sections were classified according to their positions along the longitudinal axis into four major brain regions, namely, the telencephalon (Tel), tectum opticum (TeO), corpus cerebelli (CC), and medulla oblongata (MO). In earlier studies conducted in the Mexican *Astyanax* morphs, it was found that region-specific differences in serotonergic signaling determine the disparate behavior between surface fish and cavefish (Elipot et al. 2013; Rétaux and Elipot 2013). In teleost, serotonergic neurons are mainly found in the hindbrain raphe nucleus and in three hypothalamic nuclei located in the anterior brain, and the latter is absent in mammals (Elipot et al. 2013) (fig. 2*A*). A larger anterior paraventricular nucleus in cavefish results in enhanced hypothalamic serotonergic signaling, which was shown to drive foraging behavior characteristic of cave dwellers (Elipot et al. 2013). On the other hand, experience-dependent downregulation of raphe serotonergic signaling was observed in the dominant fish among a school of surface fish (Rétaux and Elipot 2013). In our brain-sectioning scheme, sections from the TeO region cut across both superior raphe and hypothalamic serotonergic projections, whereas sections from CC and MO regions comprise mainly inferior raphe serotonergic projections.

**Fig. 3.**
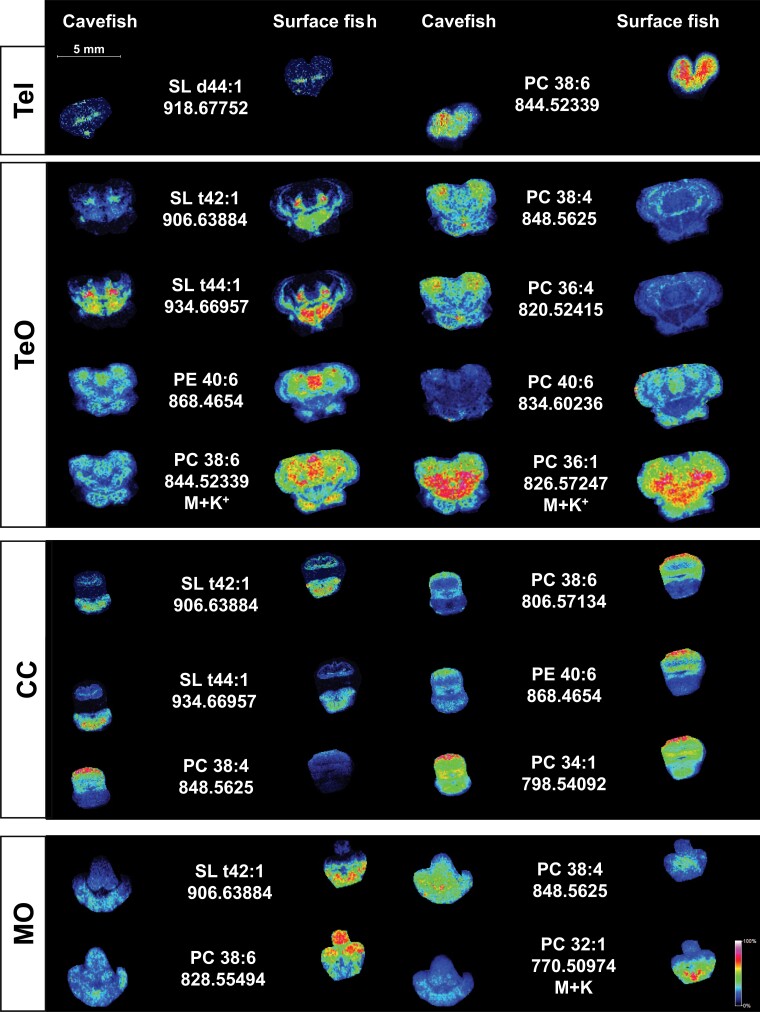
Mass spectrometric imaging of spatial lipid distribution across four brain regions in cavefish and surface fish. Frozen fish brain tissues were sectioned at 10 µm thickness, and images were acquired using a Bruker solariX mass spectrometer equipped with a 9.4 T superconducting magnet operating in the positive ion mode. The intensity of colors corresponds to lipid abundances as illustrated by the intensity bars. Scale bar: 5 mm. The *m*/z of protonated parent ions illustrated were used for data acquisition, unless otherwise indicated. M + K^+^ refer to potassium adducts. Tel, telencephalon; TeO, tectum opticum; CC, corpus cerebelli; MO, medulla oblongata.

**Fig. 4.**
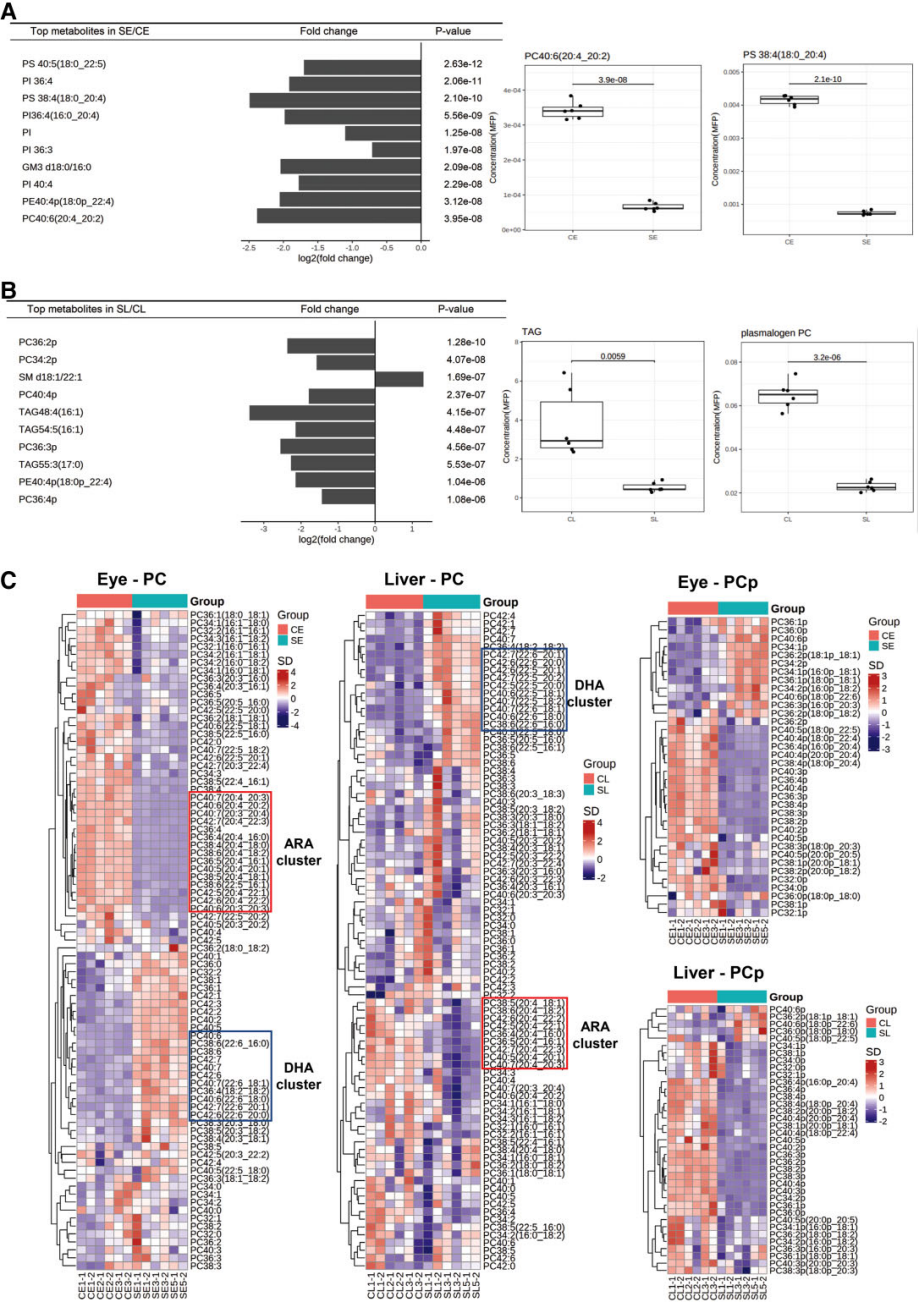
Lipidome changes in the whole-eye and whole-liver of cavefish and surface fish. (*A*) and (*B*) Differential metabolite plots illustrates top significantly different lipids between the eyes and livers of surface fish relative to cavefish. Lipids were ranked by *P*-value and log_2_(fold-changes) were plotted. In the eye, the levels of arachidonyl (20:4)-phospholipids such as PC40:6(20:4_20:2) and PS 38:4(18:0_20:4) were significantly higher in cavefish than surface fish. In the liver, total TAG and plasmalogen PC were significantly higher in cavefish than surface fish. SE, surface fish eye; CE, cavefish eye; SL, surface fish liver; CL, cavefish liver; PC, phosphatidylcholines; PS, phosphatidylserines; TAG, triacylglycerols. *P*-values from Welch’s *t*-tests were illustrated. *n* = 2 technical replicates from three cavefish and surface fish, respectively. (*C*) Heatmaps with hierarchical clustering of lipid species were plotted for the classes of PC and plasmalogen PC (PCp) in the eye and liver of cavefish and surface fish. Notable clusters of PCs containing arachidonic acids (C20:4; ARAs) and docosahexaenoic acids (C22:6, DHAs) were boxed up in red and blue, respectively. DHA-clusters were enriched in the surface fish eye and liver, whereas ARA-clusters were enriched in the cavefish eye and liver. In addition, most PCps were increased in the cavefish liver relative to the surface fish liver. *n* = 2 technical replicates from three cavefish and surface fish, respectively. SE, surface fish eye; CE, cavefish eye; SL, surface fish liver; CL, cavefish liver; PC, phosphatidylcholines; PCp, plasmalogen phosphatidylcholines.

Quantitative lipidomics revealed reductions in membrane phospholipids containing ARAs in surface fish relative to cavefish consistently across all four brain regions (fig. 2*B* and *C* and supplementary table S3, Supplementary Material online). Enriched ARA-phospholipids in cavefish brain was in agreement with GSEA analysis based on whole-brain transcriptome, indicating an enhanced ARA metabolism (fig. 1*B*). In contrast, other lipid classes exhibited region-specific changes that became evident only from region-specific lipidomics. For example, acylcarnitine levels in TeO and CC of cavefish were markedly elevated compared with surface fish (fig. 2*C* and *D*). Since acylcarnitines supply fatty acids to the mitochondria for oxidative phosphorylation (Jones et al. 2010), their higher levels in TeO and CC of cavefish suggest that these denote the primary brain regions adapted to enhanced oxidative phosphorylation compared with surface fish. Of interest, we noticed that the levels of free docosahexaenoic acids (DHAs), contrary to ARA, were significantly reduced in TeO and CC of cavefish compared with surface fish (Fig. 2*D*). Major DHA-phospholipids including PC 38:6, PC 40:6, and PE 40:6 were also reduced in the TeO and CC regions of cavefish relative to surface fish (supplementary fig. 2, Supplementary Material online). In accordance with results from quantitative lipidomics, spatial MS-imaging (MSI) of brain sections illustrated that ARA-phospholipids, such as PC 36:4 and PC 38:4 (phosphatidylcholines, PCs; see table 1 for a comprehensive list of lipid name abbreviations used in-text), were consistently increased in cavefish sections from TeO, CC, and MO regions (Fig. 3). On the other hand, DHA-phospholipids, such as PC 40:6, PC 38:6, and PE 40:6, were markedly reduced in cavefish compared with surface fish. The enrichment in DHA-phospholipids were more pronounced in surface fish TeO sections, and localized increases in DHA-phospholipids were particularly evident in the medial division of the valvula cerebelli that comprises Purkinje cells (Wullimann et al. 1996). Indeed, knockout of a member of the major facilitator superfamily, *Mfsd2a*, demonstrated to be a transporter of DHAs across the blood–brain barrier into the brain of mice, led to a significant loss of Purkinje cells in the cerebellum (Nguyen et al. 2014) (fig. 3). DHAs, therefore, are likely critical to cerebellar functions in surface fish that might have diminished importance in the evolution of cavefish. The enrichment in DHA-phospholipids extended to the eye and liver of surface fish relative to cavefish, with prominent clusters of DHA-PCs being present at higher levels in surface fish (fig. 4*C*). As for ARA-phospholipids, we noticed that their enrichment in cavefish was localized at the periventricular gray zone of the degenerated optic lobes (Wullimann et al. 1996) (fig. 3). The preferential accumulations of ARA- over DHA-phospholipids were validated also in the whole-eyes and whole-liver samples of cavefish relative to their surface-dwelling counterparts (fig. 4 and supplementary tables S5 and S6, Supplementary Material online). ARA-containing phospholipids, including phosphatidylserine PS 38:4(18:0_20:4) and phosphatidylinositol PI 36:4(16:0_20:4), were among the top significantly different lipids found in higher levels in cavefish eyes (CE) relative to surface fish eyes (SE) (fig. 4*A*). Distinct enrichment in clusters of ARA-PCs was noted in both the eye and liver of cavefish relative to surface fish (fig. 4*C*). Previous investigation conducted in zebrafish identified free ARAs released by cytoplasmic phospholipase A2 (cPLA2) cleavage of ARA-phospholipids as modulators of retinotectal arbor growth dynamics. In particular, inhibition of tectal cPLA2 (i.e., increased esterified ARAs) impedes the growth of retinal axonal arbor leading to retinotectal unsharpening (Leu and Schmidt 2008). Thus, the selective sequestration of ARAs in membrane phospholipids in cavefish may drive neuroplasticity underlying the loss of vision in the evolution of cavefish, since a neural map of an external environment of perpetual darkness is no longer essential to survival. Furthermore, the degeneration of the eyes and loss of vision also conserve energy to maximize survival in cave environment. In addition, drugs targeting the ARA-cascade, which inhibit the release of free ARAs from membrane phospholipids and lower the subsequent production of eicosanoids, have long been used as mood stabilizers in the treatment of human bipolar disorder (Rao et al. 2008). Sequestration of ARAs in neural membrane phospholipids of cavefish may, therefore, partly explain its attenuated aggressive behavior.

**Table 1.** List of Lipid Name Abbreviations.

Lipid Name Abbreviation	Lipid Name
**CE**	Cholesteryl esters
**Cho**	Free cholesterols
**TAG**	Triacylglycerols
**DAG**	Diacylglycerols
**FFA**	Free fatty acids
**acylcarnitine**	Acylcarnitine
**PC**	Phosphatidylcholines
**PCp**	Plasmalogen phosphatidylcholines
**PE**	Phosphatidylethanolamines
**PS**	Phosphatidylserines
**PI**	Phosphatidylinositols
**PA**	Phosphatidic acids
**PG**	Phosphatidylglycerols
**BMP**	bis(monoacylglycerol)phosphate
**LPC**	lyso-PC
**LPE**	lyso-PE
**LPS**	lyso-PS
**LPA**	lyso-PA
**LPI**	lyso-PI
**SL**	Sulfatides
**SM**	Sphingomyelins

### Marked Accumulation of Neutral Lipids and Plasmalogens in the Liver of Cavefish

Besides a differential accumulation of ARA- relative to DHA-phospholipids, we also observed a notable accumulation of neutral lipids triacylglycerols (TAGs) in the liver of cavefish relative to surface fish. In addition to an enhanced level of storage TAGs, cavefish liver also displayed elevated levels of plasmalogen phosphatidylcholines (PCp). The preferential accumulation of energy-dense lipids particularly in the adipose tissues of cavefish was previously reported by others (Xiong et al. 2018; Minchin 2020), which was postulated to serve as an “energy insurance” to buffer against episodes of food deprivation in caves. Cavefish were found to possess elevated body fat that enables them to sustain extended period of nutrient scarcity. Because of a lack of photosynthesis-driven primary producers in caves, cave dwellers experience prolonged nutrient limitation and are largely dependent on energy inputs from external sources including seasonal floods and bat droppings to thrive (Wilkens et al. 2000). In agreement with our observation on *Sinocyclocheilus* cavefish, fatty livers were also previously observed in the Tinaja cave form of *Astyana mexicanus* (Riddle et al. 2018).

### Cavefish Underwent Demyelination in Their Raphe Serotonergic Neuronal Populations

**Fig. 5.**
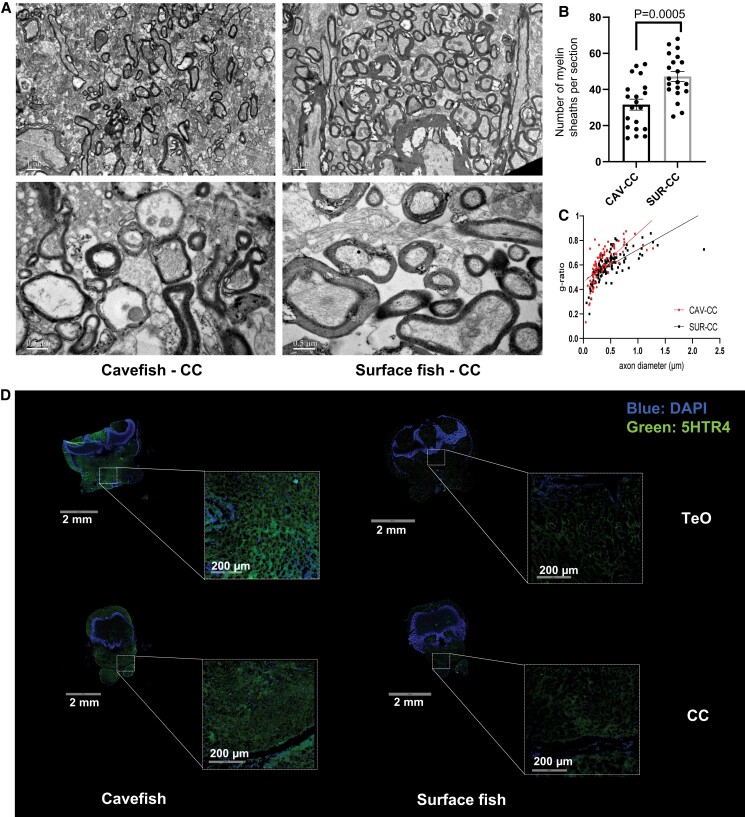
Demyelination in raphe serotonergic neurons in cavefish. (*A*) TEM images taken from the CC region of the brain of cavefish and surface fish. Scale bar = 1 µm for the top panel and 0.5 µm for the bottom panel. A greater number of myelin sheaths around individual axons and thicker myelin sheath wrapping around larger axons were observed in the CC region of surface fish relative to cavefish. Representative images from two to three independent experiments were shown. *n* = 20 sections from two cavefish and three surface fish, respectively. CC, corpus cerebelli. (*B*) Barplot illustrates the number of myelin sheaths captured per section taken from the CC region of cavefish (CAV-CC) and surface fish (SUR-CC), respectively. *n* = 20 sections from two cavefish and three surface fish, respectively. *P*-value from Welch’s *t*-test was indicated. CC, corpus cerebelli. (*C*) A plot of g-ratios against axon diameters captured in TEM images taken from the CC region of cavefish and surface fish. *n* = 88 axons from two cavefish and *n* = 132 axons from three surface fish, respectively. *P*-value from Chow’s test was used to test the statistical significance between the true coefficients in the two linear regressions constructed based on data sets from the CC regions of cavefish (CAV-CC) and surface fish (SUR-CC). CC, corpus cerebelli. (*D*) Confocal images on the distribution of serotonin receptor 4 (5-HTR4) in the TeO and CC regions of cavefish and surface fish. Representative images were from two independent experiments. Primary scale bar: 2 mm; inset scale bar: 200 µm. TeO, tectum opticum; CC, corpus cerebelli.

Based on region-specific lipidomics, we noted that sulfatides (SLs) were significantly elevated in the TeO, CC, and MO regions of surface fish relative to cavefish (fig. 2*B–D*). SL, together with its metabolic precursor galactosylceramide (GalCer), constitutes the predominant lipid components that ensure the normal structural and functional attributes of myelin sheath (Coetzee et al. 1996). MSI revealed that SL-enrichment (SL t42:1, SL t44:1) in surface fish TeO (fig. 3) was localized at the posterior tuberculum abundant in neuronal projections from the superior raphe serotonergic populations (Lillesaar 2011). In contrast, the dorsal zone of the periventricular hypothalamus of TeO sections that transverse the hypothalamic serotonergic neurons carried no observable signals of SLs but were, instead, enriched in DHA-phospholipids (PE 40:6, PC 40:6, and PC 38:6) (fig. 3). Similarly, SL-enrichment was observed in the area of the intermediate reticular formation in CC and MO sections from surface fish relative to cavefish (fig. 3), which are rich in projections from the inferior raphe serotonergic neurons (Lillesaar et al. 2009). Immunostaining of myelin distribution, that is, red fluorescent signals from myelin basic protein (supplementary fig. 3, Supplementary Material online) was in agreement with MSI data. Myelin distribution spatially overlapped with SL signals in regions corresponding to the inferior raphe serotonergic neurons in CC and the superior raphe serotonergic neurons in the TeO but not in the dorsal periventribular hypothalamus containing hypothalamic serotonergic neurons (supplementary fig. 3, Supplementary Material online). We then validated our observations based on lipidomics and immunostaining by imaging axon myelination using transmission electron microscopy (TEM) (fig. 5). We selected the hindbrain CC region for TEM because this region contains predominantly inferior raphe serotonergic innervations but not the hypothalamic serotonergic innervations. Representative TEM images from CC region revealed a greater number of myelin sheaths around individual axons captured in the visual field (fig. 5*A*), as well as a greater thickness of myelin sheaths around larger axons (fig. 5*B* and *C* and supplementary table S7, Supplementary Material online), in surface fish relative to cavefish. The g-ratio, given by the ratio of axon diameter to the summed diameter of axon and its surrounding myelin sheath, is an indicator of the degree of myelination in myelinated axons. A smaller g-ratio indicates a higher degree of myelination (Buckley, et al. 2010; Niu, et al. 2021). A plot of g-ratios against axon diameters revealed that axons with larger diameters possessed significantly thicker myelination (i.e., smaller g-ratios) in the CC region of surface fish compared with cavefish (fig. 5*C*). Based on a combination of MSI, immunostaining and TEM images examining the extent of myelination, therefore, it appears that the raphe serotonergic neuronal populations, predominantly in the hindbrain region, were more heavily myelinated in *Sinocyclocheilus* surface fish relative to cavefish. The raphe serotonergic neurons in cavefish possibly underwent appreciable demyelination in the course of their evolution. Immunostaining also revealed that cavefish possessed higher levels of 5-HT receptor 4 (5-HTR4) (green fluorescence) in both TeO and CC regions compared with surface fish (fig. 5*D*), indicating enhanced 5-HT signaling in cavefish was in agreement with previous report (Elipot et al. 2013). The dorsal periventricular hypothalamus exhibited appreciable 5-HTR4 signals indicating the presence of hypothalamic serotonergic populations in both fish species (fig. 5*D*), unlike the posterior tuberculum; however, myelin signal was absent in this region (supplementary fig. 3*A*, Supplementary Material online). Our results cumulatively suggest that relative to surface fish, cavefish undergo demyelination specifically in their raphe serotonergic neurons but not hypothalamic serotonergic neurons.

### Gene Expressions Underlying Environment-Specific Metabolic Adaptations in Fish Brain and Liver

**Fig. 6.**
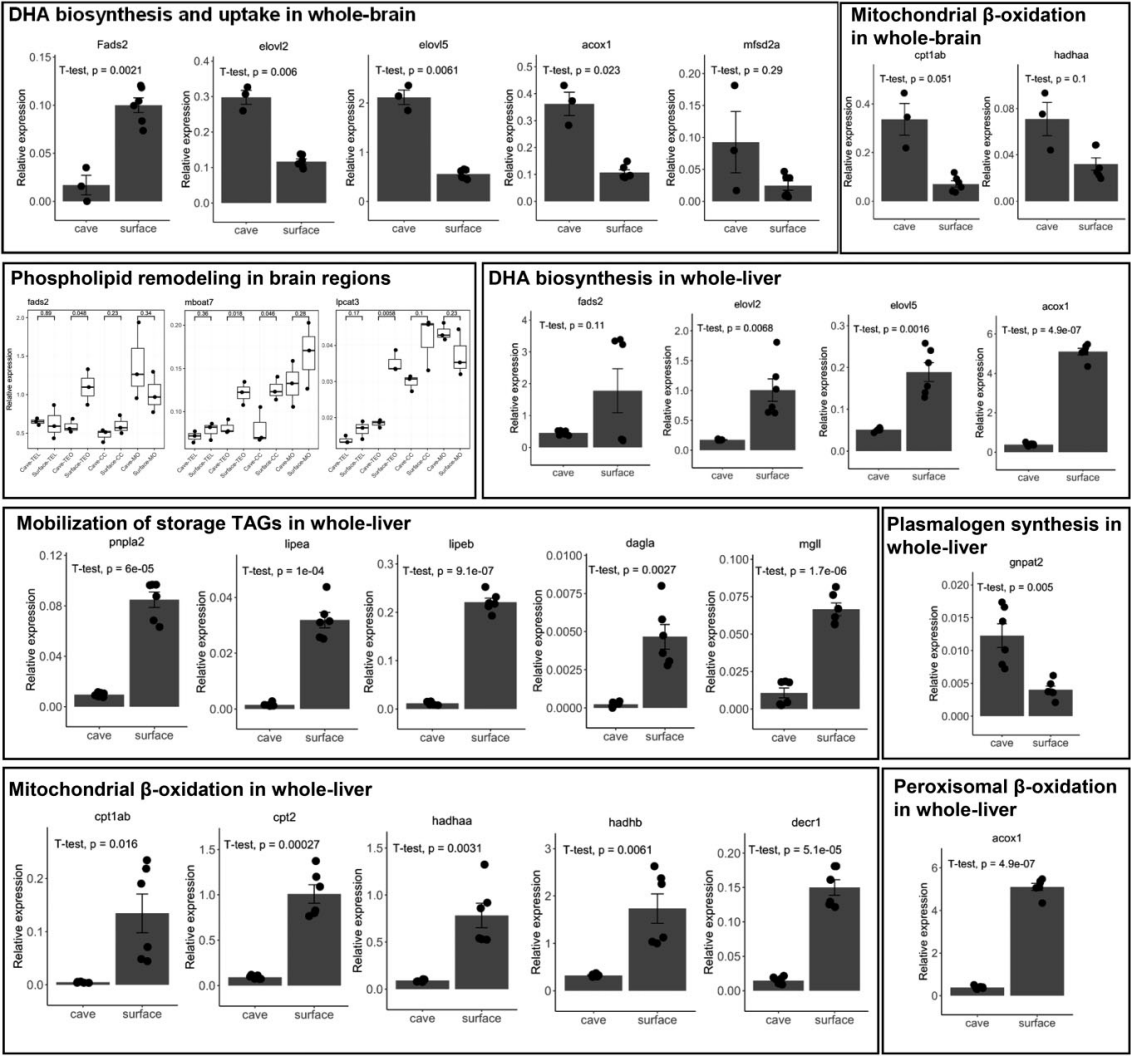
mRNA expression of genes in the brain and liver involved in the metabolic adaptations of cavefish and surface fish to their distinct habitats. Relative expression values of genes were normalized to those of β-actin. Barplots display changes in the relative expression of genes in the whole-brain or whole-liver of cavefish and surface fish. Boxplots illustrate changes in relative expression of genes in cavefish and surface fish across the four regions of the brains. For barplots, *n* = 3 technical replicates from one cavefish and two surface fish, respectively. For boxplots, *n* = 3–4 brain sections from three biological replicates for Tel, TeO, and CC, *n* = 2 brain sections from three biological replicates for MO. In barplots, means ± SEM were plotted. In boxplots, the median is indicated by the horizontal line and the first and third quartiles were represented by the box edges. The lower and upper whiskers extend from the hinges to the smallest and largest values, respectively, with individual samples indicated as dots. In all plots, *P*-values from Welch’s *t*-test were indicated. *fads2*: *fatty acid desaturase 2*; *elovl2*: *elongation of very-long-chain fatty acids 2*; *elovl5*: *elongation of very-long-chain fatty acids 5*; *acox1*: *acyl-coenzyme A oxidase 1*; *mfsd2a*: *member of the major facilitator superfamily 2a*; *cpt1ab*: *carnitine-palmitoyltransferase 1a or 1b*; *hadhaa*: *hydroxyl-coenzyme A dehydrogenase alpha subunit*; *mboat7*: *membrane bound-O-acyltransferase domain containing 7*; *lpcat3*: *lysophosphatidylcholine acyltransferase 3*; *pnpla2*: *patatin-like phospholipase domain containing 2*; *lipea*: *lipase, hormone-sensitive a*; *lipeb*: *lipase, hormone-sensitive b*; *dagla*: *diacylglycerol lipase*, *alpha*; *mgll*: *monoglyceride lipase*; *gnpat2*: *glyceronephosphate O-acyltransferase 2*; *cpt2*: *carnitine-palmitoyltransferase II*; *decr1*: *2,4-dienoyl CoA reductase 1, mitochondrial*. Tel, telencephalon; TeO, tectum opticum; CC, corpus cerebelli; MO, medulla oblongata.

In our lipidomic investigation of the brain, the eye and liver of cavefish versus surface fish, we discovered four essential differences in lipid metabolism between the two fish species, which might relate to the differential selection pressures of their distinct habitats. Relative to surface fish, cavefish exhibited 1) enhanced oxidative phosphorylation in the brain; 2) preferential accumulation of DHA-phospholipids over ARA-phospholipids in the brain, eye, and liver; 3) accumulation of fat (in the form of storage TAGs) and plasmalogens PC in the liver; and (4) selective demyelination of raphe serotonergic neurons in its hindbrain. To elucidate the candidate genes underlying the differential lipid metabolism between cavefish and surface fish, we examined the relative expressions of several genes along the pathways of DHA biosynthesis, uptake and phospholipid remodeling, fat mobilization, mitochondrial and peroxisomal β-oxidation, as well as plamalogen biosynthesis in the brain and liver (fig. 6, supplementary table S8, Supplementary Material online). DHA biosynthesis in teleosts can proceed either via the Δ4 desaturase pathway or the Sprecher pathway (Oboh et al. 2017; Matsushita et al. 2020). The biosynthesis of DHAs involves fatty acid desaturases (fads) and elongation of very-long-chain fatty acid (Elovl) proteins. Unlike mammals that carry both *fads1* and *fads2* genes, virtually all teleosts possess only the *fads2* gene but with varying copy numbers. *fads2* has acquired diverse functions during the process of teleost evolution and is able to introduce double bonds at different positions along the fatty acyl chains (Xie et al. 2021). Elovls catalyze the two-carbon elongation of preexisting fatty acyl moieties. Elovl2 displays substrate preference for C20 and C22 polyunsaturated fatty acids (PUFAs), and plays a significant role in the biosynthesis of DHAs via the Sprecher pathway (Monroig et al. 2018). Our qRT-PCR showed that in the brain, *fads2* expression was significantly higher in surface fish relative to cavefish but not for other genes along the Sprecher pathway (*elovl2*, *elovl5*, and *acyl-coenzyme A oxidase 1*: *acox1*). In contrast, while the levels of *fads2* in the liver were not significantly different between cavefish and surface fish, other genes of the Sprecher pathway (*elovl2*, *elovl5*, and *acox1*) were significantly elevated in the liver of surface fish compared with cavefish. Based on our results, it appears that surface fish increases DHA biosynthesis in the brain via the Δ4 desaturase pathway but instead relies on the Sprecher pathway to enhance DHA biosynthesis in the liver. In addition, the level of *mfsd2a* in the brain, which mediates DHA uptake from the systemic circulation into the brain (Nguyen et al. 2014), was not significantly different between cavefish and surface fish. Hence, surface fish likely retains a local, neurological supply of DHAs to sustain critical brain functions, instead of relying on DHA uptake from the systemic circulation. Indeed, it was reported that among different tissues, the teleost brain possesses the highest expression of *fads2*, and this enriched pattern of expression in marine fish brain serves to retain a local, functional Δ6 desaturase to ensure a sufficient supply of DHAs to neural tissues (Monroig et al. 2018). The enhanced biosynthesis of DHAs was particularly evident for the TeO region of the surface fish’s brain, which may be related to the essential roles of DHAs in facilitating advanced functions of photoreceptors in the eye (Crawford et al. 1999). In addition to augmented local biosynthesis of DHAs, increased incorporation of DHAs into membrane phospholipids via remodeling processes were also evident in the brain. The relative expressions of lysophospholipid acyltransferases with substrate specificity for PUFAs, including *membrane bound-O-acyltransferase domain containing 7* (*mboat7*) and *lysophosphatidylcholine acyltransferase 3* (*lpcat3*) (Hishikawa et al. 2008; Zarini et al. 2014), were significantly elevated in surface fish particularly in the TeO region (fig. 6).

Corroborating our lipidomic observations, cavefish brain exhibited marginally elevated expressions of genes mediating mitochondria β-oxidation, such as the *carnitine-palmitoyltransferase 1a or 1b* (*cpt1ab*) and *hydroxyl-coenzyme A dehydrogenase alpha subunit* (*hadhaa*) compared with surface fish. Contrary to the brain, the liver of surface fish displayed markedly elevated levels of genes involved in fat mobilization (*pnpla2*: *patatin-like phospholipase domain containing 2*; *lipea*: *lipase, hormone-sensitive a*; *lipeb*: *lipase, hormone-sensitive b*; *dagla*: *diacylglycerol lipase*, *alpha*; *mgll*: *monoglyceride lipase* that mediate the breakdown of TAGs to diacylglycerols DAGs to monoacylglycerols MAGs and, finally, to free fatty acyls), as well as those involved in mitochondrial β-oxidation (*cpt1ab*, *hadhaa*, *cpt2*: *carnitine-palmitoyltransferase II*, and *decr1*: *2,4-dienoyl CoA reductase 1*) and peroxisomal β-oxidation (*acox1*) (fig. 6). Thus, fatty livers in cavefish might have resulted from attenuated mobilization and oxidation of fatty acyls compared with surface fish. In addition, cavefish liver also exhibited enhanced expression of *glyceronephosphate O-acyltransferase 2* (*gnpat2*) (fig. 6), the rate-limiting enzyme in peroxisomal matrix governing plasmalogens biosynthesis (Brites et al. 2004), in agreement with the observed increases in PCps in cavefish liver.

We also attempted to investigate the expressions of genes mediating SL biosynthesis and breakdown in the hindbrain regions of *Sinocyclocheilus*, including galactose *3-O-sulfotransferase 4* (*gal3st4*) and *arylsulfatase A* (*arsa*), but the levels of gene expression were too low to render reliable quantitative comparisons between cavefish and surface fish. Other yet unknown genes candidates might possibly regulate the changes in myelination observed in the hindbrains of *Sinocyclocheilus* that warrant further investigation in future studies.

## Discussion

As cavefish thrive in nutrient-poor environments under perpetual darkness, obtaining food becomes a top priority that determines its survival (Xiong et al. 2018). In the process of adapting to their cave habitats, for example, the Mexican *Astyanax* has evolved various behavioral changes including the loss of aggression and schooling and swim randomly to facilitate foraging and maximize their chances of coming across food (Elipot et al. 2013). As a result, multiple independent cave species in physically separated caves evolved similar behavioral and morphological traits. Molecular mechanisms underlying such parallelism or convergence in evolution have remained largely unclear (Gross 2012). Our lipid-centric investigation has identified 1) enhanced oxidative phosphorylation in cavefish brain, 2) reduced biosynthesis of DHAs in the brain, eye, and liver of cavefish, 3) accumulation of fat and plasmalogens in cavefish liver, as well as 4) the selective demyelination of raphe serotonergic neurons in cavefish as the prominent lipid pathways possibly underlying behavioral adaptations to caves over freshwater environments.

**Fig. 7.**
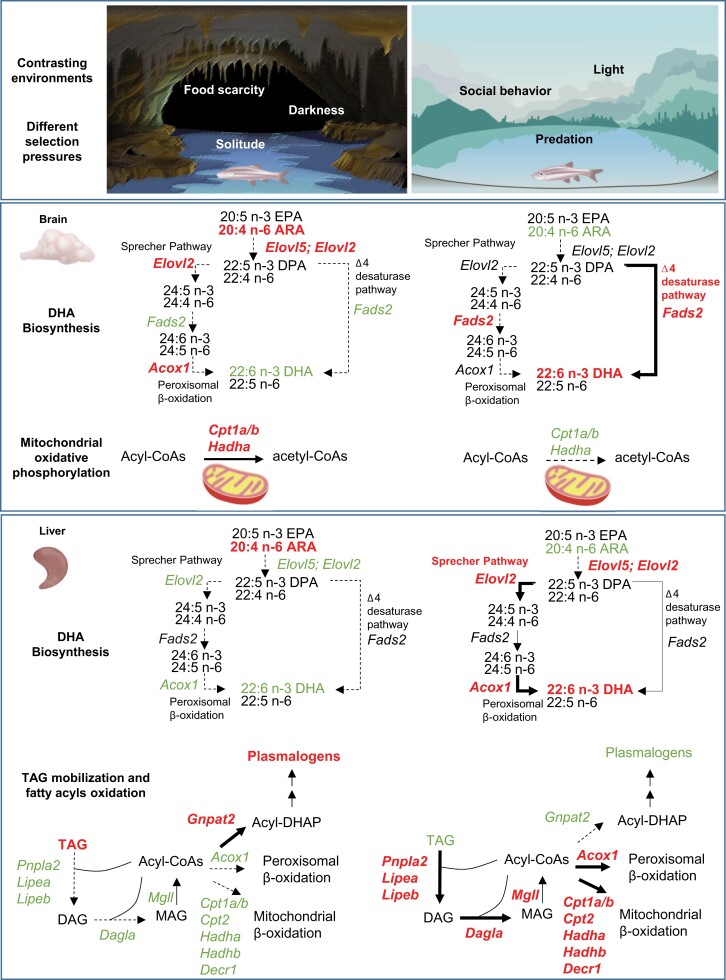
Schematic diagram summarizes major pathways in the brain and liver of cavefish and surface fish underlying metabolic adaptations to their distinct environments as revealed by lipidomics and qRT-PCR. The cave environment entails a solitary life marked by food scarcity and darkness under which resource utilization and minimizing energy expenditure become the major determinants of extended survival. Surface fish, in comparison, is subjected to an environment of light and constant predation and developed social behaviors including schooling and aggressive dominance to reduce risk of predation. Our lipidomics and qRT-PCR supported reduced DHA biosynthesis along the Δ4 desaturase pathway and enhanced oxidative phosphorylation in the brains of cavefish as the prominent neurological pathways contributing to troglomorphic adaptations. Outside the brain, a global reduction in DHA biosynthesis along the Sprecher pathway coupled with attenuated mobilization of fat storage and reduced β-oxidation in the mitochondria and peroxisomes denotes key metabolic changes underlying troglomorphic adaptions that primarily serve to reduce energy expenditure. *fads2*: *fatty acid desaturase 2*; *elovl2*: *elongation of very-long-chain fatty acids 2*; *elovl5*: *elongation of very-long-chain fatty acids 5*; *acox1*: *acyl-coenzyme A oxidase 1*; *mfsd2a*: *member of the major facilitator superfamily 2a*; *cpt1ab*: *carnitine-palmitoyltransferase 1a or 1b*; *hadhaa*: *hydroxyl-coenzyme A dehydrogenase alpha subunit*; *mboat7*: *membrane bound-O-acyltransferase domain containing 7*; *lpcat3*: *lysophosphatidylcholine acyltransferase 3*; *pnpla2*: *patatin-like phospholipase domain containing 2*; *lipea*: *lipase, hormone-sensitive a*; *lipeb*: *lipase, hormone-sensitive b*; *dagla*: *diacylglycerol lipase*, *alpha*; *mgll*: *monoglyceride lipase*; *gnpat2*: *glyceronephosphate O-acyltransferase 2*; *cpt2*: *carnitine-palmitoyltransferase II*; *decr1*: *2,4-dienoyl CoA reductase 1, mitochondrial*.

Region-specific quantitative lipidomics and spatial MSI revealed reduced DHA biosynthesis and incorporation into membrane phospholipids in cavefish brains relative to surface fish, which led us to postulate that DHAs likely facilitate cerebellar functions in surface fish that are no longer pivotal to survival in cavefish. In freshwater ecosystems, schooling behavior reduces the risk of predation (Magurran 1990). DHA-enriched diet was previously shown in larval yellowtail *Seriola* to affect the volumetric growth of TeO and CC regions important to visual acuity and swimming performance. In particular, the onset of schooling behavior was closely matched with the time-point at which significant volumetric expansion in TeO and CC was observed (Ishizaki et al. 2000). Similarly in human cohorts, increased maternal DHA intake from seafood consumption was positively associated with improvements in prosocial behavior and fine motor skills of their children (Hibbeln et al. 2007). The loss of DHA-enriched neural domains might also be partly explained by vision loss in cavefish driven by an environment of total darkness, since DHA represents the PUFA uniquely utilized by photoreceptors (Crawford et al. 1999). We then validated via qRT-PCR that the specific reductions in membrane DHAs in the brain of cavefish were attributed to reduction in DHA biosynthetic capacity via *fads2* along the Δ4 desaturase pathway (fig. 7) coupled with a diminished remodeling of polyunsaturated DHAs into membrane phospholipids mediated by *mboat7* and *lpcat3*. Indeed, *fads2* was identified as a key metabolic gene that increases survival on DHA-deficient diets within freshwater ecosystems (Twining et al. 2016)—a pivotal factor to the colonization of freshwater habitats in fishes (Ishikawa et al. 2019). Kitano and colleagues demonstrated that additional copy number of *fads2* in Pacific Ocean sticklebacks contributes to survivorship on DHA-deficient freshwater habitats and that freshwater stickleback populations with longer evolution history in freshwater are associated with higher *fads2* copy numbers (Ishikawa et al. 2019). *Sinocyclocheilus* ancestors may have acquired enhanced biosynthetic capacity of DHAs during the course of evolution. The elevated incorporation of DHAs into neural and optic membrane lipids enhance vision and facilitate neural execution of complex behavioral traits, such as schooling, important to the survival of surface fish in freshwater habitats. In comparison, food scarcity replaces predation as the major determinant of survivorship in cave habitats; coupled with the total darkness in the cave environments that *S. anophthalmus* reside, the maintenance of vision and complex social behavior such as schooling diminished in importance relative to minimizing energy expenditure.

In addition to the brain, cavefish also experienced similar reductions in DHA lipids in its liver—a key organ governing systemic lipid metabolism. In contrast to the brain, the reduction of DHA biosynthesis in cavefish liver results from an attenuated flow along the Sprecher pathway (fig. 7). This observation suggests that the loss of DHA biosynthetic capacity probably has evolutionary significance outside of the brain. The membrane pacemaker theory of metabolism proposes the central role of DHA as a major contributor to the degree of cellular membrane polyunsaturation, which, in turn, governs the molecular activities of membrane ion pumps that determine the basal metabolic rate of an organism (Hulbert 2007). For example, a strong positive correlation was observed between the DHA content of phospholipids and the heart rate of a diverse group of mammals ranging from mice to whales (de Duve and Hayalshl 1978). DHA content of membranes was found to increase the molecular activity of sodium/potassium ATPase pumps that denote a major contributor to basal metabolic rate (Turner et al. 2003). Cavefish are known to possess lower energy expenditure and requirement relative to surface fish, in order to withstand prolonged period of nutrient deprivation in an environment with infrequent food supply. For example, the degeneration of the eyes, which have high energy requirement, allows cavefish to conserve up to 15% of energy (Krishnan and Rohner 2017), whereas the loss of a functional circadian rhythm saves another 27% of energy (Moran et al. 2014). Thus, based on the membrane pacemaker theory of cellular metabolism, a global reduction in membrane DHAs in *Sinocyclocheilus* cavefish may serve to lower basal metabolic rate and conserve energy to maximize survival in a nutrient-limiting environment. On a similar note, we also observed that cavefish greatly elevate fat storage in the liver, possibly via suppressed breakdown of storage TAGs into fatty acyl constituents and reduced oxidation of fatty acyls through the mitochondria and peroxisomes (fig. 7). The reductions in fatty acyls oxidation are in agreement with a lower energy requirement and reduced basal metabolic rate. Cavefish was postulated to have evolved compensatory mechanisms that enable them to remain physiologically healthy, with comparable lifespan and without appreciable accumulation of advanced glycation end-products (AGEs) compared with surface fish; in spite of deleterious diabetes-related pathologies characterized by excess fat accumulation (i.e., fatty livers), insulin resistance and hyperglycemia that serve to cope with irregular food supply (Riddle et al. 2018). In this aspect, it is worthy to note that plasma levels of plasmalogen PCs were negatively correlated with the abundances of AGEs in human patients (Zhang et al. 2017). Plasmalogens, by virtue of their vinyl ether linkages, can counter the generation of reactive oxygen species induced by AGEs (Braverman and Moser 2012). It remains an interesting question whether the upregulation of plasmalogen biosynthesis denotes a metabolic adaptation to cope with excess fat accumulation in cavefish.

Finally, a selective demyelination of raphe serotonergic neurons might be attributed to social isolation of cavefish ancestors and a loss in requirement for neural plasticity that shapes complex social behavior, since it can be potentially energetically costly to maintain oligodendrocytes needed for myelination (Harris and Attwell 2012). Indeed, demyelination had been identified as a contributing factor of cognitive impairment resulting from social isolation in humans and rodent models (Baraban et al. 2016). Our observation of enhanced myelination specifically at raphe serotonergic neurons in surface fish provides a plausible explanation on why only surface fish, but not cavefish, are able to execute experience-dependent downregulation of raphe serotonergic signaling in establishing social dominance. Indeed, preliminary results from our resident-intruder assay indicate that surface fish exhibit a markedly higher level of aggressive dominance compared with cavefish (supplementary fig. S4, Supplementary Material online). Regulation of myelination based on social experience can alter the intensity of raphe serotonergic signaling. Experience-dependent shaping of myelination to achieve neural plasticity had also been demonstrated in adult human brains (Tomassy et al. 2016). Our current work, however, did not identify the gene candidates central to the regulation of raphe serotonergic neuron myelination in *Sinocyclocheilus*.

## Conclusion

Through a combination of quantitative lipidomics with spatial MSI, we revealed that neural lipid metabolic plasticity, in particular enhanced oxidative phosphorylation, reduced DHA biosynthesis and membrane incorporation, and demyelination of raphe serotonergic neurons might contribute to neuroplasticity underlying troglomorphic behavioral adaptations, that is, loss of aggressive dominance and schooling in the evolution of cavefish. Given that the gross morphological anatomy of the brains between the two fish species are largely conserved apart from degeneration of the optic lobes in cave-dwelling fish, our study illustrates how regional compartmentalization in metabolism and distribution of lipids can alter neural plasticity to modulate behavior during the course of evolution. On top of neurological adaptations, we uncovered that systemic metabolic adaptations, such as the development of fatty livers, global reductions in membrane DHAs, and the accretion of liver plasmalogens, also contribute to troglomorphic adaptations to a cave habitat. The central theme of troglomorphic adaptations entails the loss of nonessential morphological and behavioral traits (regressive evolution) in order conserve energy in an environment of limiting, irregular food supply, as proper resource allocation becomes the major determinant of survival in a cave habitat. The selection pressure for these troglomorphic adaptations in turn drives parallel or convergent evolution in cave dwellers. Notwithstanding these findings, our study has numerous limitations. First, our study results do not allow the interpretation of causality. Although the investigated genes may underlie changes in lipid metabolic pathways that are differentially regulated between cavefish and surface fish, functional studies and the generation of genetic knockouts are imperative to determine whether these genes cause the metabolic changes that contribute to the evolution of troglomorphic traits in *Sinocyclocheilus*. Such experimental validations are currently infeasible in *Sinocyclocheilus*, largely circumscribed by the rarity of this genus, long growth term, and the recent classification of *Sinocyclocheilus* as second-class protected animals in China. We also failed to elucidate the key genes regulating the myelination of raphe serotonergic neurons in *Sinocyclocheilus*, owing to the low expression levels of our candidate genes investigated (*gal3st4*, *arsa*). A functional characterization of *fads2* in different tissues of *Sinocyclocheilus*, for example, using heterologous expression system in yeast (Matsushita et al. 2020), which can help determine whether qualitative changes in fatty acid metabolic enzymes in addition to gene expressions may modulate the biosynthetic capacity of DHAs, was not conducted in this study. In addition, it will be meaningful to investigate in future studies the upstream evolutionary genetic basis for the altered expressions of genes underlying the differences in behavioral and morphological adaptations between the two fish species reported in this study.

## Materials and Methods

### Animals

Collection sites for cavefish (*S. anopthalmus*) and surface fish (*S. angustiporus*) were near the city of Kunming, Yunnan province (fig. 1*A*). The orange mark indicates collection location of cavefish at Jiuxiang cave N 25.05478°, E 103.37975°. Surface fish were collected from Huangnihe River in Agang Town (green marks), Luoping, at N 25.00905°, E 103.59256°. After collection, fish were allowed to recover overnight in laboratory tanks to minimize metabolic distress due to transport, then euthanized with 0.05% tricaine methanesulfonate, and dissected within 2 days with no feeding in between. The dorsal surface of the head was dissected away to expose the brain, the fresh brains were carefully dissected from the head and frozen on the dry ice for MSI. For immunohistochemistry, the brains were fixed in 4% paraformaldehyde overnight at 4 °C and equilibrated in 30% sucrose prior to cross-cryosectioning. All experimental procedures involving animals were conducted and approved by the Animal Care and Use Committee of the Institute of Zoology, Chinese Academy of Sciences (approved protocol: IOZ18002).

### Brain Tissue Sectioning

Frozen fish brain tissues were fixed in position on the cutting stage. All tissues were sectioned at 10 µm thickness using a Leica CM1950 cryostat (Leica Microsystems GmbH, Wetzlar, Germany) at −18 °C and mounted onto indium tin oxide-coated glass slides (Type I 0.7 mm/100ea, HST Inc., Newark, NJ, USA). The slide was put into a vacuum desiccator and dried for approximately 1 h. For analysis of lipids, a mixture (250 µl) of AgNPs and DHB (1 mg/ml AgNPs and 10 mg/ml DHB in ACN/H_2_O 8:2) solution was sprayed on the tissue section using an in-house electrospray-based matrix deposition device and at a solvent flow rate of 800 µl/h (Guan et al. 2018). The glass slides were dried in the vacuum desiccator for approximately 1 h prior to analysis on MALDI MS-Imaging (MSI).

### Lipid Extraction

Lipids were extracted from whole-brains/brain sections using a modified Bligh and Dyer’s protocol as previously described (Lam et al. 2021). Tissues were homogenized in extraction solvent, that is, chloroform: methanol 1:2 (v/v) containing 10% MilliQ water on a bead ruptor (OMNI, USA). Following incubation, 350 µl of MilliQ water and 250 µl of ice-cold chloroform were added to induce phase separation. Samples were then centrifuged at 16, 260 × g at 4 °C for 5 min. The lower organic phase containing lipids were transferred to a new tube. The extraction was repeated once via addition of another 500 µl of ice-cold chloroform to the remaining aqueous phase, and the extractions were pooled and dried in a SpeedVac under the organic mode.

### HPLC-MS/MS Quantitative Lipidomics

Dried lipid extracts were resuspended in chloroform: methanol (1:1) prior to LC-MS/MS analysis (Song et al. 2020). Analyses of polar lipids in whole-brain and brain sections were conducted on Exion-UPLC coupled with Sciex 6500 Plus QTRAP that runs on Analyst 1.6.3, whereas neutral lipids were analyzed on an Agilent 1260 HPLC connected to Sciex 5500 QTRAP. Analyses were conducted in the electrospray ionization mode, using the following source parameters, 5500: CUR 10, CAD High, TEM 350 °C, GS1 35, GS2 35; 6500: CUR 10, CAD High, TEM 400, GS1 20, GS2 20. Internal standard cocktail used for normalization of lipidome data in whole-brain and brain sections included DMPC, DMPE, PA-C17:0, d_31_-PS, DMPG, C14:0-BMP, SL d18:1/17:0, CL22:1(3)-14:1, LPC 17:0, LPS 17:1, LPA 17:0, LPE 17:1, SM d18:1/12:0, Cer d18:1/17:0, GluCer d18:1/8:0, d_3_-16:0-carnitine from Avanti Polar Lipids, PI-8:0/8:0 was purchased from Echelon Biosciences, whereas d_31_-FFA 16:0 and d_8_-FFA-20:4 were obtained from Sigma-Aldrich and Cayman Chemicals, respectively. Analyses of lipidomes from whole-eye and whole-liver samples of cavefish and surface fish were conducted on a Shimadzu Nexera X2 LC-30AD UPLC coupled with Sciex Triple Quad 7500. Analysis was performed in the electrospray ionization mode with the following source parameters: Ion Source Gas 1 35, Ion Source Gas 2 70, Curtain Gas 32, Temperature 450 °C, CAD Gas 9. Internal standard cocktail for normalization of the eye and liver lipidome data included d_9_-PC32:0(16:0/16:0), d_9_-PC36:1p(18:0p/18:1), d_7_-PE33:1(15:0/18:1), d_9_-PE36:1p(18:0p/18:1), d_31_-PS(16:0/18:1), d_7_-PG33:1(15:0/18:1), d_7_-PI33:1(15:0/18:1), d_7_PA33:1(15:0/18:1), C14-BMP, d_8_-SM d18:1/18:1, SL-d18:1/17:0, d_7_-LPC 18:1, d_7_-LPE 18:1, LPA-C17:0, LPI-C17:1, LPS-C17:1, LPG-C17:1, DAG(16:0/16:0)-d_5_, and DAG(18:1/18:1)-d_5_ obtained from Avanti Polar Lipids; TAG(14:0)_3_-d_5_, TAG(16:0)_3_-d_5_, TAG(18:0)_3_-d_5_, d_6_-CE18:0, and d_6_-Cho purchased from CDN isotopes; and d_3_-16:0-carnitine from Cambridge Isotope Laboratories. d_31_-FFA-16:0 from Sigma-Aldrich and d_8_-FFA-20:4 from Cayman Chemicals were used for quantitation of saturated/monounsaturated fatty acids and PUFAs, respectively.

### MALDI-FITCR MSI

MALDI-FTICR mass spectrometric analysis of the tissue sections were performed using a Bruker solariX mass spectrometer equipped with a 9.4 T superconducting magnet (Li et al. 2016). Data were collected in the positive ion mode, in broadband over a mass range of 100–1,200 m/z with a resolution of 200,000 at m/z 200; mass calibrations were performed externally using sodium trifluoroacetate (NaTFA), using 150 shots per scan by a Smart Beam II laser operating at 150 Hz, a laser focus of 50 µm. For MALDI MSI analysis, the entire tissue section was analyzed averaging 1 scan per spectrum (per pixel) with fixed raster step size (Tel region: 70 µm; TeO region: 85 µm; CC region: 80 µm; MO region: 50 µm). All the data were processed using DataAnalysis 4.0 (Bruker Daltonics) and FlexImaging 3.0 sofware (Bruker Daltonics).

### Immunohistochemistry

Fish brain sections were incubated in PBS with 0.5% Triton-100 for permeabilizing. Antigens were unmasked by microwaving sections in 10 mM citrate buffer, pH 6.0, 5 min. Then blocked in 5% donkey serum in PBS for 1 h at room temperature and incubated overnight at 4 °C with the rabbit antiserotonin antibody (1:2000, Sigma No. S5545), the rabbit anti-5HT4 (1:100, Abcam No. ab60359), and rat antimyelin basic protein (1:200, Abcam No. 7349). Brain sections were washed three times with PBS for 5 min and incubated with the secondary antibodies (Alexa 488 conjugated or Alexa-568 conjugated, 1:1000, Thermo Fisher Scientific) at room temperature for 1 h. Images were required by Leica Aperio Versa 200.

### Transmission Electron Microscopy and g-Ratio Analyses

The corpus cerebelli (CC) region of the brain from cavefish and surface fish were dissected and immediately fixed with 2.5% glutaraldehyde overnight and postfixed with 1% osmium tetroxide for 1 h at 4^ ^°C. Samples were then stained with 3% uranyl acetate for 30 min at room temperature, washed with deionized water five times for 10 min each round, and dehydrated in a series of acetone treatments and infiltrated in embed-812 resin. The embedded tissues were cut into 70 nm slices and observed using a transmission electron microscope (TEM) (JEM 1400) at 80 kV. The number of myelin sheaths in individual TEM sections was counted, and g-ratios were calculated as the diameter of the axon divided by the diameter of the axon and its surrounding myelin sheath using ImageJ (Buckley et al. 2010; Lam et al. 2021).

### Relative Gene Expression Using qRT-PCR

Total RNAs were extracted from brain sections using 1 ml of TRIzol reagent (Invitrogen, Cat No: 15596-026). For each sample preparation, 0.2 ml of chloroform was added and the aqueous phase was collected in fresh tubes and then mixed with 0.5 ml of isopropyl alcohol for pelleting RNA. To remove DNA contamination, RNA pellets were washed with 75% ethanol, digested with DNase for 30 min, and then pelleted via addition of sodium acetate and lithium chloride. RNA pellets were then washed in 75% ethanol and reconstituted in nuclease-free water. cDNA was synthesized using the iScript cDNA Synthesis Kit (Bio-Rad, Cat No 1708891). qRT-PCR was performed using the SYBR Green PCR kit (Bio-Rad, Cat No 1725120) on a Bio-Rad CFX Connect 384-Real-Time PCR Detection machine. Relative expression values of genes were normalized to those of β-actin. Gene-specific primers used for amplification were listed in table 2.

**Table 2.** Gene-specific Primers for qRT-PCR Analyses.

Gene	Primer (5′–3′)
**elovl2-F**	TTGTCAACCATTCCATCCAT
**elovl2-R**	CCAACCTTAGCAAGTCTGT
**elovl5-F**	GTCCAGTTTGTCCTGACCAT
**elovl5-R**	CCGTTTATTGATCCGTTCGAG
**hadhaa-F**	CAGCAGCCAGTAAGAGAC
**hadhaa-R**	CCACGACTATGATGACCTT
**hadhb-F**	CAGGAAGTGAAGACCAGTAA
**hadhb-R**	TGAGTGACAGCATCGTCT
**decr1-F**	CCACCACTCTGTCTTCAC
**decr1-R**	CCGTTCAGCACTATGTCA
**acox1-F**	CAGCAAGAGCAATGAGGA
**acox1-R**	TGAAGGGCATAAAGCAGAG
**mgll-F**	CCTCCAGCACATTGACAT
**mgll-R**	ATCCCGTGAGACCCATTT
**dagla-F**	CATCCTGTCCTTCCTCCT
**dagla-R**	AGCAATRAATCCGCCACTT
**lipea-F**	GTGGTCATCAGTATGGTGT
**lipea-R**	TGGAAGCAATCCTTGTCAA
**lipeb-F**	GCAAGGCGTCTGAATACT
**lipeb-R**	AGTGTGATGTCAAGGATGG
**pnpla2-F**	CTTCACCAACACCTCCATT
**pnpla2-R**	ACTCTTCCTCAGCCTCTC
**gnpat2-F**	TGAAGTTGAGTCGTATGAGT
**gnpat2-R**	TCTGGCGTCTACACTGAT
**fads2-F**	TCGGACACTATGCTGGAGAAG
**fads2-R**	GATGACCGAAGTCATGCTGC
**mfsd2a-F**	ACTGAGCAGAAGGAGAGG
**mfsd2a-R**	CACCAAGGAACAAGACCAT
**cpt1ab-F**	ACCAGTCAGACTCCTCTC
**cpt1ab-R**	TTGCTGGATATGTGGAAGTT
**cpt2-F**	GCAATGGGTCAAGGCTTTG
**cpt2-R**	GAAACCATCAGGCACCAC
**lpcat3-1F**	CATCAGCTGCTGGCTAATCAC
**lpcat3-1R**	GTGCCAGAYCGCCAGGAATA
**mboat7-1F**	CCGCTACCTAAGTCCTCCAG
**mboat7-1R**	AGACAGAGCAGGRGCACTTC
**β-actin-F**	GAAGATCAAGATCATTGCTCCC
**β-actin-R**	ATGTCATCTTGTTCGAGAGGT

### Statistical Analysis

To examine changes in lipidome composition, lipids in whole-brains were expressed in µmol/g dry mass, whereas lipids in individual brain sections were expressed in µmoles/section. To examine changes in membrane lipidomes, lipid levels in whole-livers and whole-eyes were expressed in molar fractions of total polar lipids, where total polar lipids denote the sum total of all phospholipids and sphingolipids detected. Hierarchical clustering using ward method (ward.D2 in R hclust) was performed on log_2_-transformed lipid levels in µmol/g dry mass. Heatmaps were drawn using ComplexHeatmap v2.7.10.9001. Clustering was visually evaluated and patterns of interests were manually selected. For analysis of lipid correlations in the brain of cavefish and surface fish, data were log-transformed and correlations between lipid pairs were calculated based on the Spearman correlation analysis using Hmisc v4.4-2. Correlation coefficient cutoff was set at ≥0.7 and *P*-value cutoff was set at *P* < 0.05. Correlations with corresponding *P* < 0.05 were visualized using chord diagrams drawn with chorddiag v0.1.2 and circlize v0.4.12, where bandwidth indicates number of correlations and color indicates direction of correlation. The blue shade indicates positive correlations, whereas the red shade indicates negative correlation between lipids. For brain sections, only two-group comparisons were made between brain sections of cavefish and surface fish obtained from the same brain region (i.e., Tel, TeO, CC, or MO) using Welch’s *t*-test. Fold change and −log_10_*P*-values were presented in volcano plots. For GSEA, a ranked gene list comparing whole-brain transcriptomes between cavefish and surface fish was obtained from Meng et al. (2013, 2018) (supplementary table S1, Supplementary Material online). GSEA was performed using gseGO function from R package clusterProfiler 3.14.3 with *P*-value cutoff set at <0.05. Statistical analyses were performed using R 4.0.2.

## Supplementary Material

msac050_Supplementary_Data
