# Supplementary material for: Quantitative Lipidomics and Spatial MS-Imaging Uncovered Neurological and Systemic Lipid Metabolic Pathways Underlying Troglomorphic Adaptations in Cave-Dwelling Fish
Source: Mol Biol Evol. 2022 Mar 12;39(4):msac050. doi: 10.1093/molbev/msac050 (PMC9011034; doi:10.1093/molbev/msac050)
Supplement: msac050_Supplementary_Data [file msac050_supplementary_data.zip › 20220302 fish MS Supplemental figures.pptx]

## Slide 1
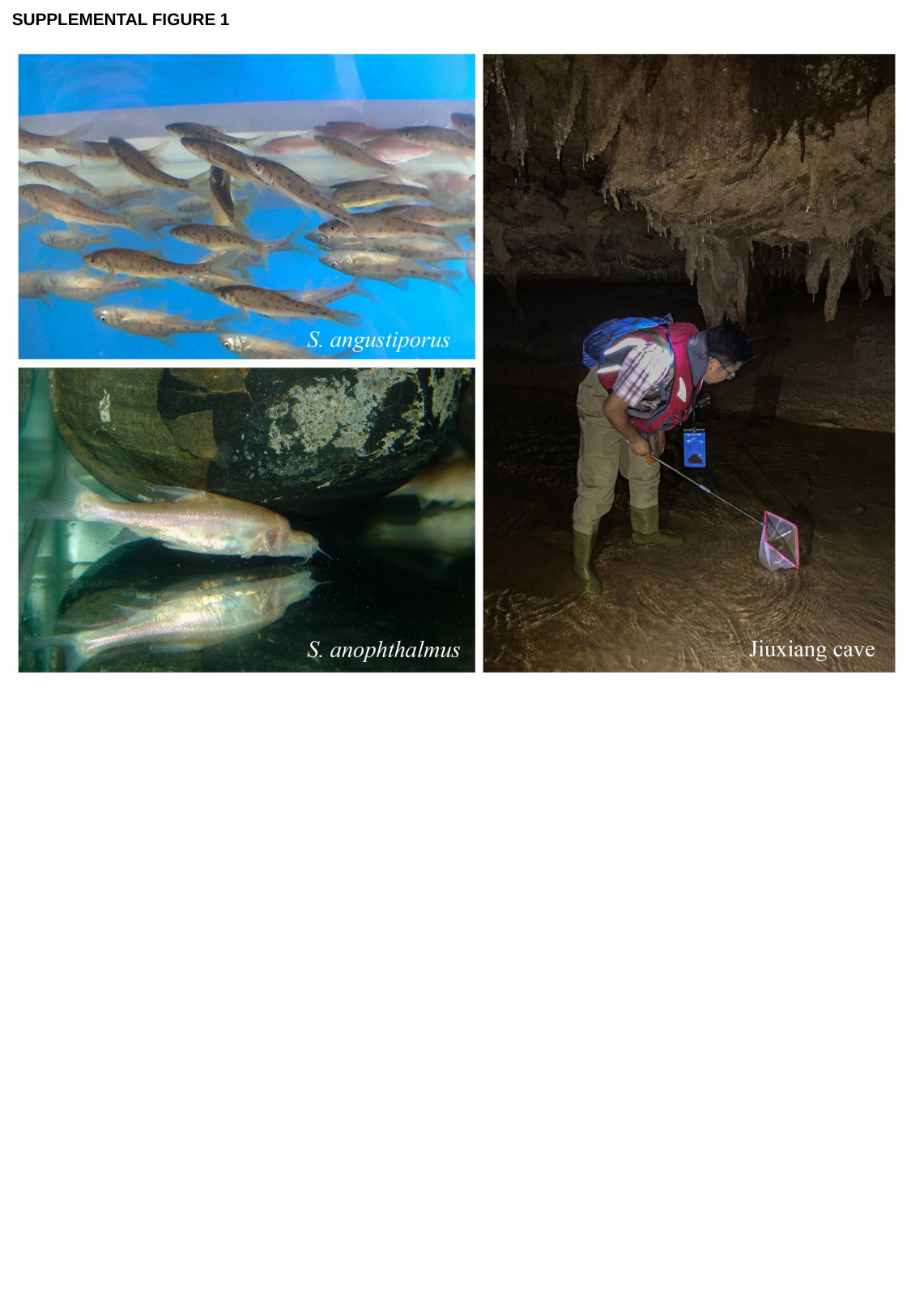

SUPPLEMENTAL FIGURE 1

## Slide 2
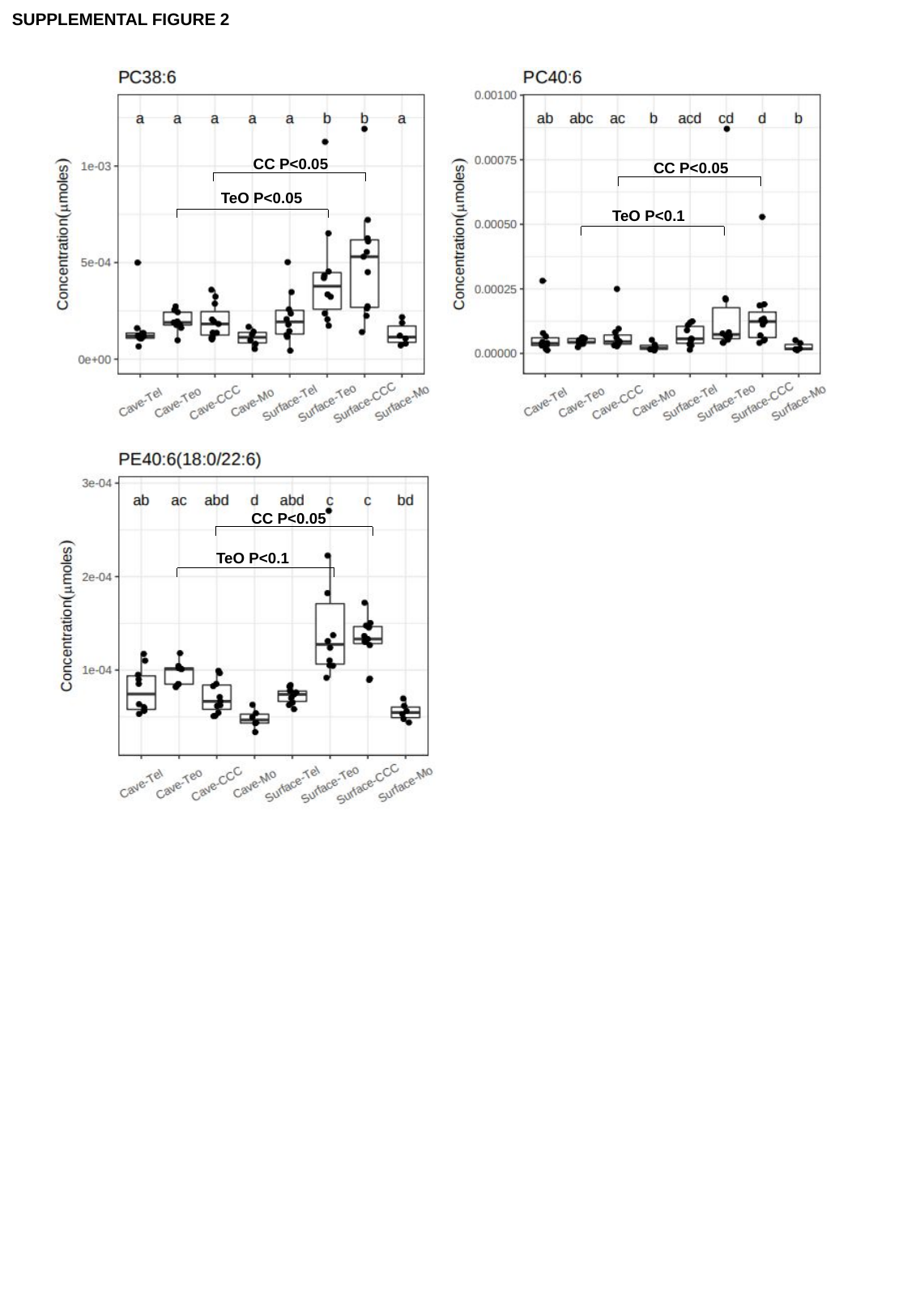

SUPPLEMENTAL FIGURE 2
CC P<0.05
CC P<0.05
TeO P<0.05
TeO P<0.1
CC P<0.05
TeO P<0.1

## Slide 3
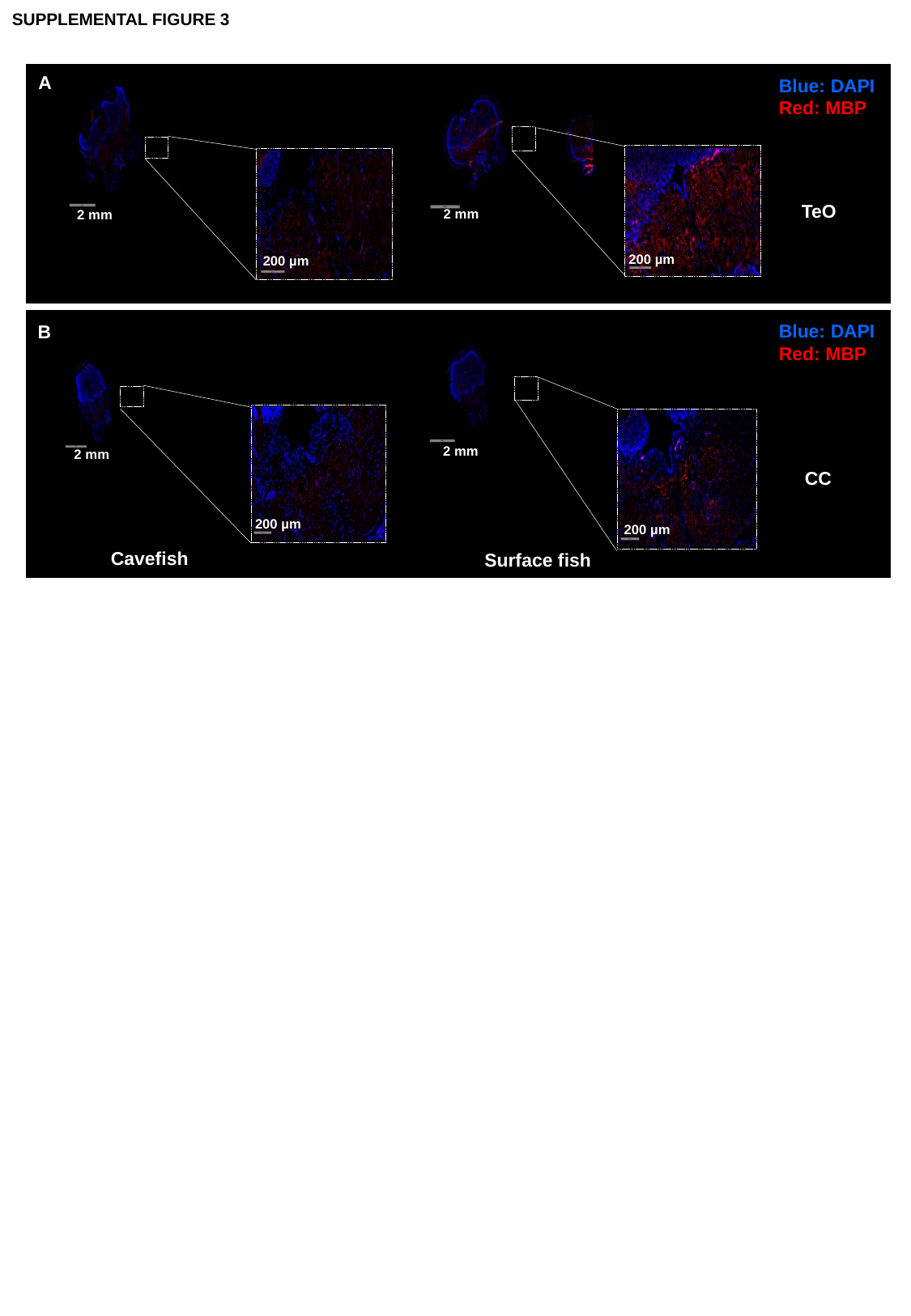

SUPPLEMENTAL FIGURE 3
A
Blue: DAPI
Red: MBP
TeO
2 mm
2 mm
200 µm
200 µm
Blue: DAPI
Red: MBP
CC
Cavefish
Surface fish
B
2 mm
2 mm
200 µm
200 µm

## Slide 4
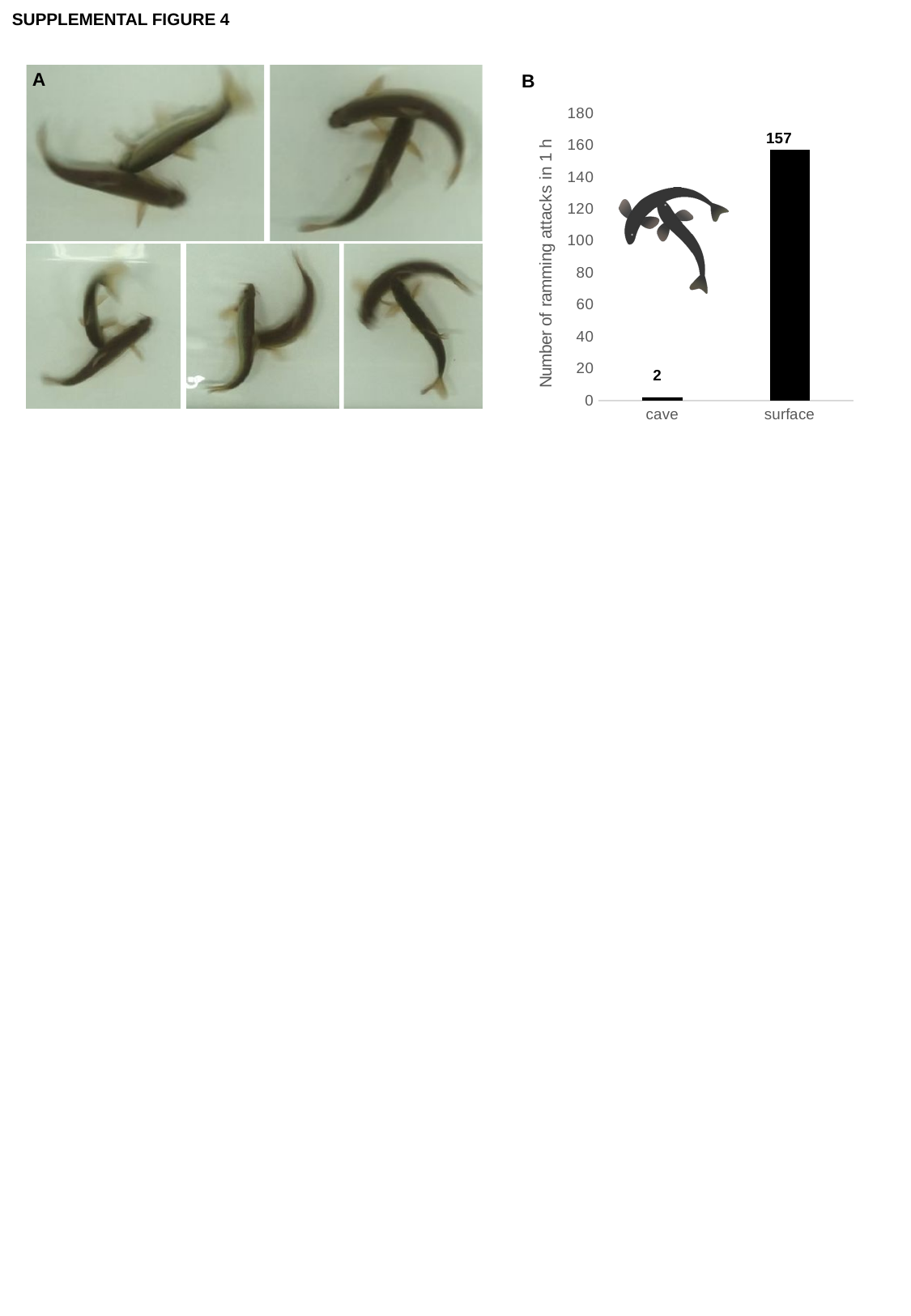

SUPPLEMENTAL FIGURE 4
A
B
### Chart
| Category | |
|---|---|
| cave | 2.0 |
| surface | 157.0 |157
2
