# Supplementary figures and images for: Quantitative Lipidomics and Spatial MS-Imaging Uncovered Neurological and Systemic Lipid Metabolic Pathways Underlying Troglomorphic Adaptations in Cave-Dwelling Fish
Source: Mol Biol Evol. 2022 Mar 12;39(4):msac050. doi: 10.1093/molbev/msac050 (PMC9011034; doi:10.1093/molbev/msac050)

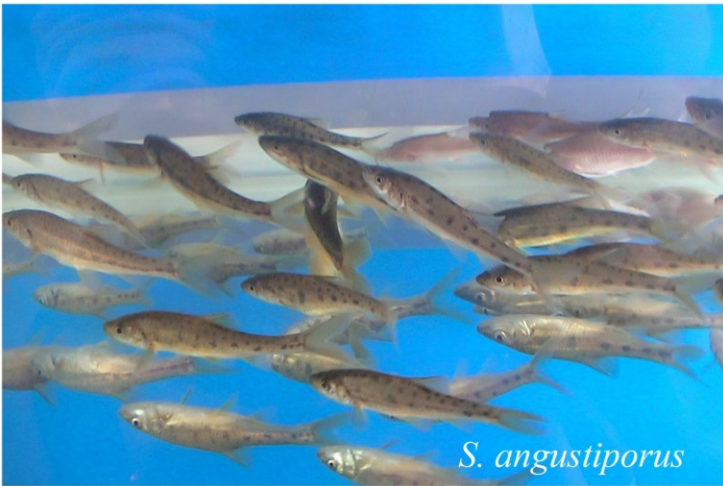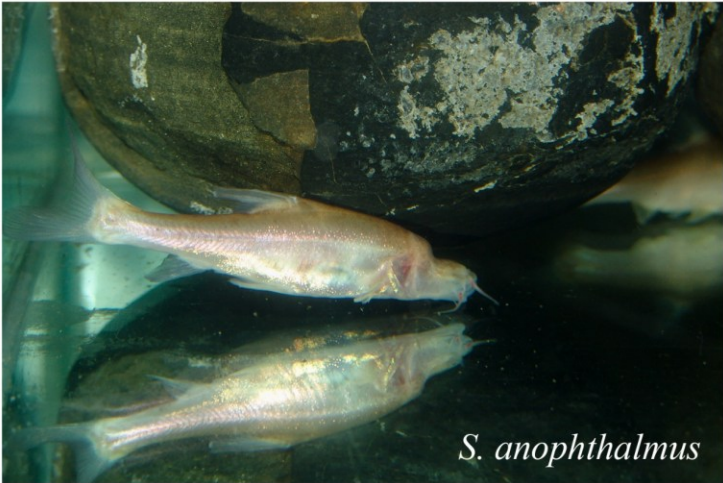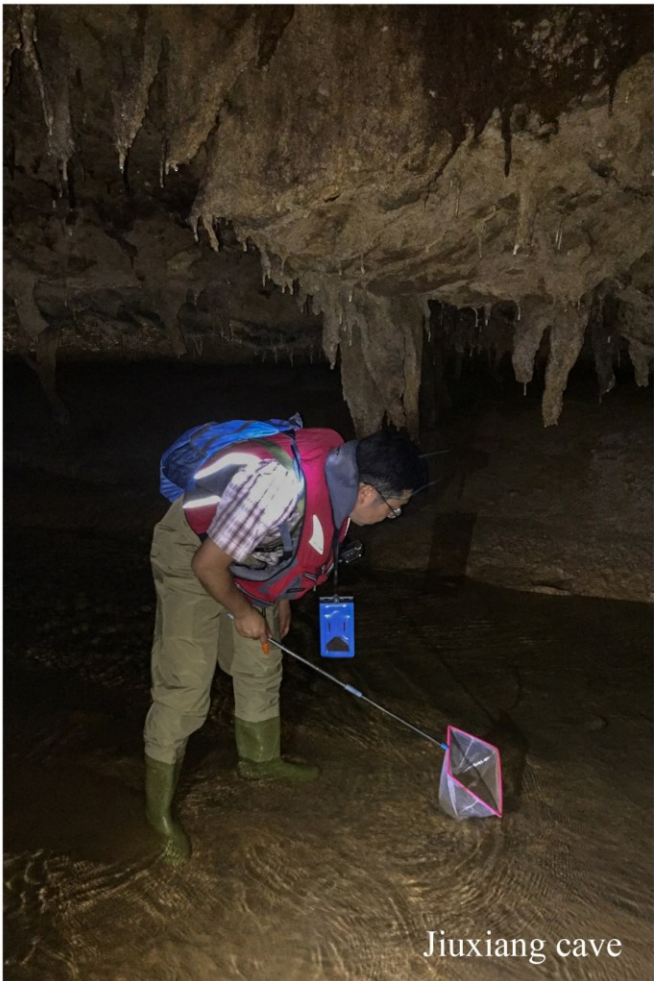

SUPPLEMENTAL FIGURE 2

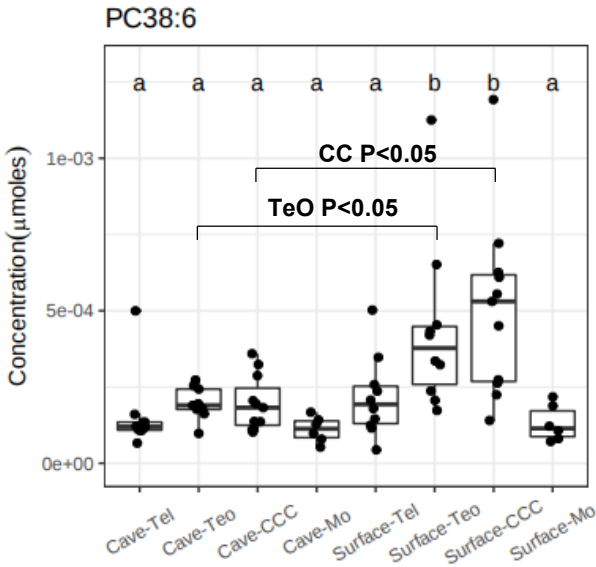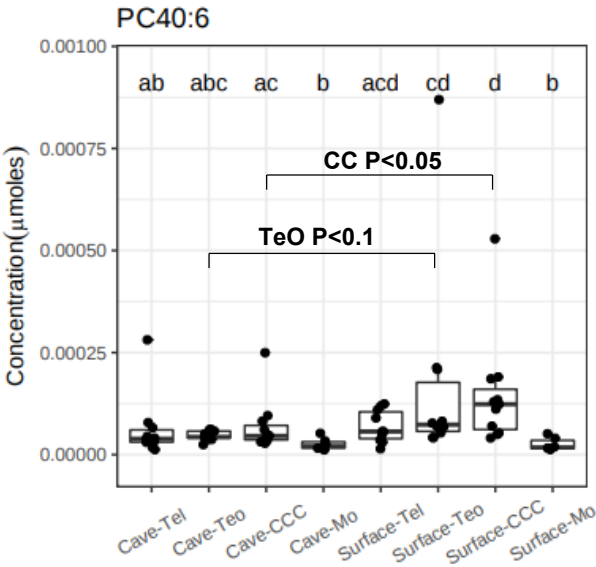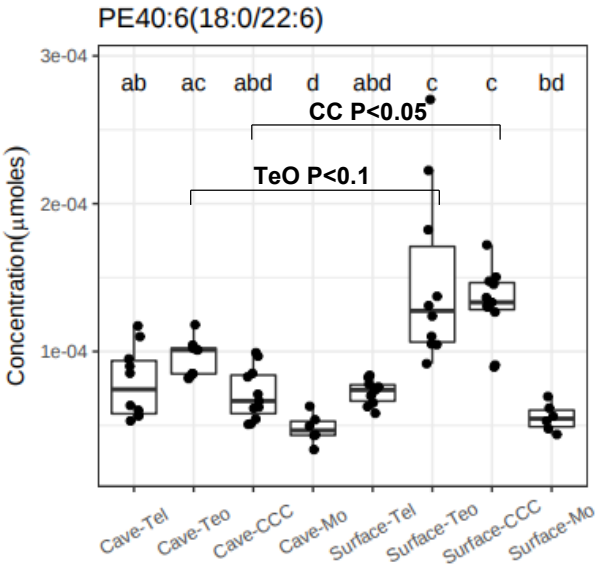

SUPPLEMENTAL FIGURE 3

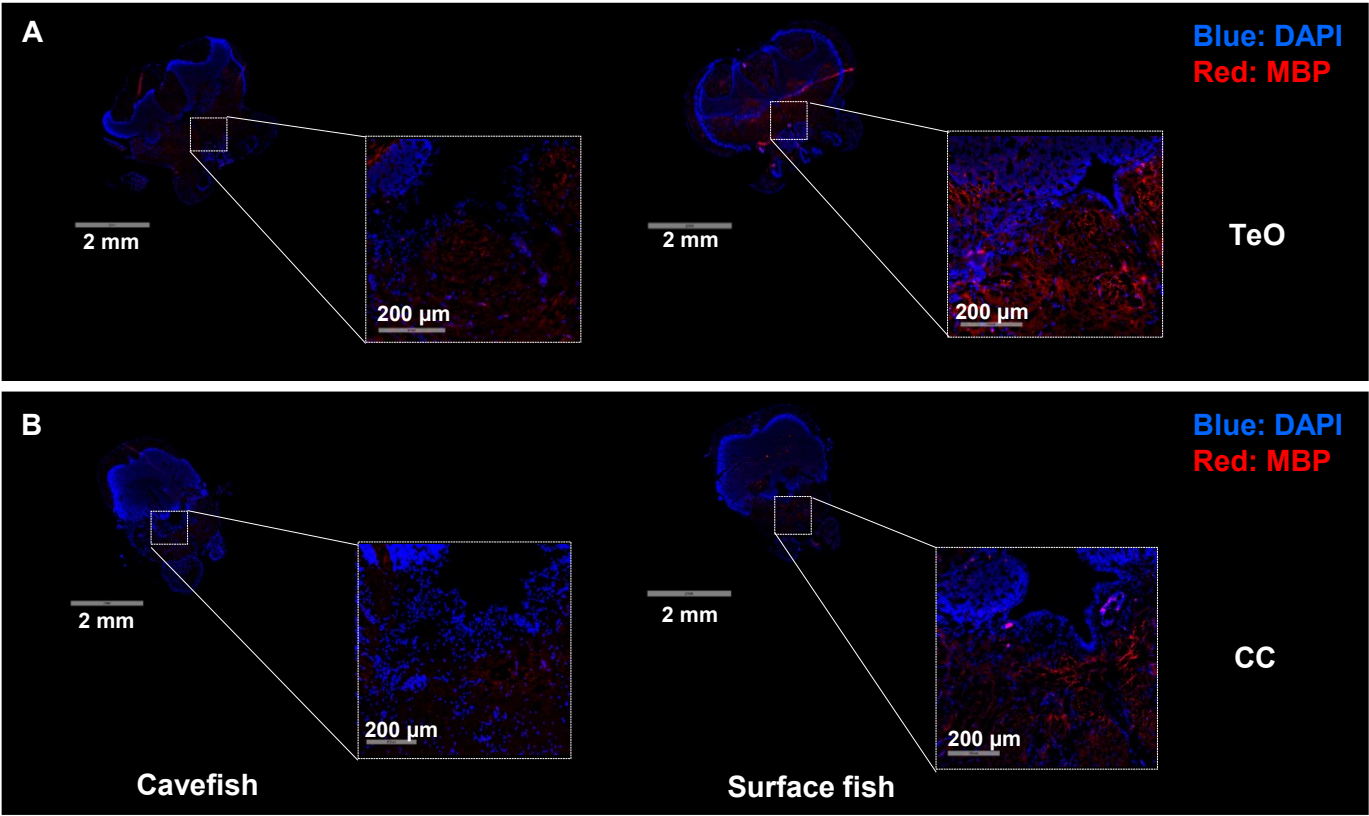

SUPPLEMENTAL FIGURE 4

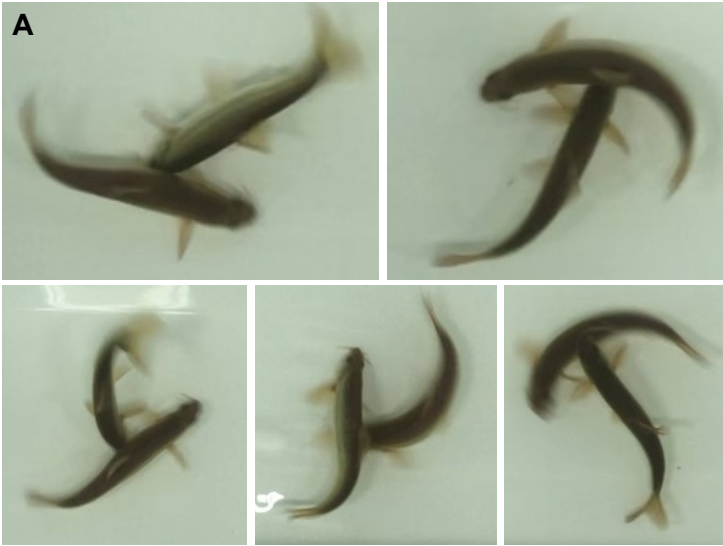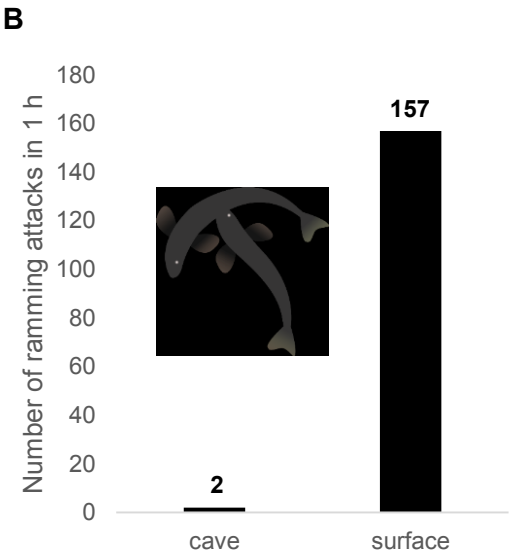

Supplement: msac050_Supplementary_Data [file msac050_supplementary_data.zip › 20220302 fish MS Supplemental figures.pdf]
